# Global disease burden attributed to low physical activity in 204 countries and territories from 1990 to 2019: Insights from the Global Burden of Disease 2019 Study

**DOI:** 10.5114/biolsport.2023.121322

**Published:** 2022-11-22

**Authors:** Achraf Ammar, Khaled Trabelsi, Souhail Hermassi, Ali-Asghar Kolahi, Mohammad Ali Mansournia, Haitham Jahrami, Omar Boukhris, Mohamed Ali Boujelbane, Jordan M. Glenn, Cain C. T. Clark, Aria Nejadghaderi, Luca Puce, Saeid Safiri, Hamdi Chtourou, Wolfgang I. Schöllhorn, Piotr Zmijewski, Nicola Luigi Bragazzi

**Affiliations:** 1 Department of Training and Movement Science, Institute of Sport Science, Johannes Gutenberg-University Mainz, Mainz, Germany; 2 Interdisciplinary Laboratory in Neurosciences, Physiology and Psychology: Physical Activity, Health and Learning (LINP2), UFR STAPS (Faculty of Sport Sciences), UPL, Paris Nanterre University, Nanterre, France; 3 High Institute of Sport and Physical Education, University of Sfax, Tunisia; 4 Research laboratory, Education, Motricity, Sport and Health (EM2S), LR15JS01, High Institute of Sport and Physical Education, University of Sfax, Tunisia; 5 Sport Science Program (SSP), College of Arts and Sciences (CAS), Qatar University, Doha 2713, Qatar; 6 Social Determinants of Health Research Center, Shahid Beheshti University of Medical Sciences, Tehran, Iran; 7 Department of Epidemiology and Biostatistics, School of Public Health, Tehran University of Medical Sciences, Tehran, Iran; 8 Ministry of Health, Manama 410, Bahrain; 9 Department of Psychiatry, College of Medicine and Medical Sciences, Arabian Gulf University,Manama 323, Bahrain; 10 Research Unit: “Physical Activity, Sport, and Health”, UR18JS01, National Observatory of Sport, Tunis 1003, Tunisia; 11 Sport and Exercise Science, School of Allied Health, Human Services and Sport, La Trobe University, Melbourne 3086, Australia; 12 Department of Health, Exercise Science Research Center Human Performance and Recreation, University of Arkansas, Fayetteville, AR 72701, USA; 13 Centre for Intelligent Healthcare, Coventry University, Coventry CV1 5FB, UK; 14 Physical Medicine and Rehabilitation Research Center, Aging Research Institute, Tabriz University of Medical Sciences, Tabriz, Iran; 15 Social Determinants of Health Research Center, Department of Community Medicine, Faculty of Medicine, Tabriz University of Medical Sciences, Tabriz, Iran; 16 Department of Neuroscience, Rehabilitation, Ophthalmology, Genetics, Maternal and Child Health (DINOGMI), University of Genoa, Genoa, Italy; 17 Tuberculosis and Lung Disease Research Center, Tabriz University of Medical Sciences, Tabriz, Iran; 18 Department of Community Medicine, Faculty of Medicine, Tabriz University of Medical Sciences, Tabriz, Iran; 19 Jozef Pilsudski University of Physical Education in Warsaw, 00-809 Warsaw, Poland; 20 Laboratory for Industrial and Applied Mathematics (LIAM), Department of Mathematics and Statistics, York University, Toronto, ON M3J 1P3, Canada

**Keywords:** Global burden of disease, Physical inactivity, Death rates, Disability-adjusted life years, Public Health

## Abstract

The purpose of this investigation is to estimate the global disease burden attributable to low physical activity (PA) in 204 countries and territories from 1990 to 2019 by age, sex, and Socio-Demographic Index (SDI). Detailed information on global deaths and disability-adjusted life years (DALYs) attributable to low PA were collected from the Global Burden of Disease Study 2019. The ideal exposure scenario of PA was defined as 3000-4500 metabolic equivalent minutes per week and low PA was considered to be less than this threshold. Age-standardization was used to improve the comparison of rates across locations or between time periods. In 2019, low PA seems to contribute to 0.83 million [95% uncertainty interval (UI) 0.43 to 1.47] deaths and 15.75 million (95% UI 8.52 to 28.62) DALYs globally, an increase of 83.9% (95% UI 69.3 to 105.7) and 82.9% (95% UI 65.5 to 112.1) since 1990, respectively. The age-standardized rates of low-PA-related deaths and DALYs per 100,000 people in 2019 were 11.1 (95% UI 5.7 to 19.5) and 198.4 (95% UI 108.2 to 360.3), respectively. Of all age-standardized DALYs globally in 2019, 0.6% (95% UI 0.3 to 1.1) may be attributable to low PA. The association between SDI and the proportion of age-standardized DALYs attributable to low PA suggests that regions with the highest SDI largely decreased their proportions of age-standardized DALYs attributable to low PA during 1990-2019, while other regions tended to have increased proportions in the same timeframe. In 2019, the rates of low-PA-related deaths and DALYs tended to rise with increasing age in both sexes, with no differences between males and females in the age-standardized rates. An insufficient accumulation of PA across the globe occurs together with a considerable public health burden. Health initiatives to promote PA within different age groups and countries are urgently needed.

## INTRODUCTION

It is well documented that regular physical activity (PA) is beneficial for health through risk-reduction of diseases and premature mortality [1, 2]. However, despite the development and implementation of country-specific PA guidelines (e.g., Great Britain, Canada, Australia, USA), as well as through the World Health Organization (WHO) and the European Union, 27.5% of adults worldwide do not accumulate sufficient PA to meet current public health guidelines [3]. The consequences of physical inactivity on disease burden have been assessed in several countries, such as Great Britain [4] and South Africa [5]. Overall, physical inactivity increases the risk of many diseases, while subsequently decreasing life expectancy [4, 5]. In a large-scale, global investigation using data from 2009 and before, Lee et al. [6] estimated that by eliminating physical inactivity, life expectancy of the world’s population is estimated to increase by 0.68 (0.41 to 0.95) years.

An up-to-date and detailed report using the best available evidence is urgently needed in order to evaluate the impact of previous policies and help influence future policy decisions. The Global Burden of Diseases, Injuries, and Risk Factors Study (GBD) is a continuous and robust effort to evaluate the disease burden of more than 80 risk factors for 204 countries and territories across the globe [7–9], and provides a unique opportunity to understand the landscape of low PA. In this study, we evaluated the impact of low PA on global disease burden in 204 countries and territories from 1990 to 2019 by age, sex, and Socio-demographic Index (SDI), using the estimates from the most recent GBD 2019.

## MATERIALS AND METHODS

### Overview and data sources

This study is part of GBD 2019, which is currently the most comprehensive and systematic report to date estimating the levels and trends of burden caused by 369 diseases and injuries, as well as 87 risk factors from 1990 to 2019. Seven super-regions, 21 regions, and 204 countries and territories were involved in GBD 2019. The general methodology of GBD 2019 and the comparative risk assessment specifically for low PA have been described in previous publications [10, 11]. In the present study, data on the disease burden attributable to low PA were extracted through a result tool available on the website of Institute for Health Metrics and Evaluation (IHME)[http://ghdx.healthdata.org/gbd-results-tool]. The original data sources used for the estimations of low PA can be found on the GBD 2019 Data Input Sources Tool website [http://ghdx.health-data.org/gbd-2019/data-input-sources]. Since no identifiable data were used in GBD 2019, a waiver of informed consent was reviewed and approved by the University of Washington Institutional Review Board.

### Definition of low physical activity

PA was quantified using total metabolic equivalent (MET) minutes per week, which was calculated by summating the frequency and duration per activity and the METs corresponding to the intensity of each activity. One MET is defined as the energy cost of sitting quietly and is equivalent to 1 kcal/kg/hour [12]. Although the accepted threshold/ definition for physical inactivity is <600 MET-minutes/week, this threshold may not capture all increased mortality risk caused by inadequate PA [13]. In GBD studies, the counterfactual level of risk exposure used is the risk exposure that is both theoretically possible and minimizes risk in the exposed population that consequently captures the maximum population attributable burden [11]. For PA in GBD 2019, best available epidemiological evidence from published and unpublished relative risks by PA level and the lowest observed PA level from cohorts were used to select a single PA exposure level that minimizes risk for all causes of deaths combined to establish the theoretical minimum-risk exposure level (TMREL) [11]. The TMREL for PA was estimated as 3000-4500 MET minutes per week, at which minimal deaths across outcomes occurred [11, 13]. Thus, low PA was defined as less than the TMREL in GBD studies.

### Analysis strategies

Random sampling studies of the general adult population (aged ≥ 25 years) that captured PA across all domains of life (leisure/ recreation, work/household and transport) were selected for the analysis. Only durations of at least ten minutes at a time were included. PA data were primarily derived from two standardized questionnaires: The Global Physical Activity Questionnaire (GPAQ) and the International Physical Activity Questionnaire (IPAQ). Other survey instruments that asked about duration, frequency, and intensity of PA performed across all activity domains were also included.

Deaths and disability-adjusted life years (DALYs) related to low PA were estimated using the GBD comparative risk assessment framework. The framework pairs low PA with known disease-specific outcomes. To be paired, sufficient evidence for a causal relationship between low PA and disease outcome is required. According to the results of pooled cohort studies [10], ischemic heart disease, ischemic stroke, type 2 diabetes mellitus, colon and rectum cancer and breast cancer were proved to be significantly associated with low PA. Disability-adjusted life-years (DALYs) were a summary that quantifies the overall health loss due to low PA and were calculated by summing years lived with disability and years of life lost. One DALY represents the loss of the equivalent of one year in full health [14]. DisMod-MR 2.1, a Bayesian meta-regression tool developed for the GBD study, was used to simultaneously derive the estimates of deaths and DALYs attributable to low PA. The proportion of deaths and DALYs attributable to low PA was estimated using population attributable fraction (PAF), which represents the corresponding proportions that would be reduced if the exposure to low PA was increased to an ideal exposure scenario. More details on the modeling strategies can be obtained elsewhere [10, 11].

### Socio-Demographic Index (SDI)

SDI was a composite indicator quantifying the development status for each location-year [10, 11]. SDI ranged from 0 (less developed) to 1 (most developed) and was calculated based on lag-distributed income per capita, mean educational attainment for individuals aged ≥ 15 years, and total fertility rate under 25 years. Based on SDI quintiles, the 204 countries and territories included in this paper were classified into five groups: low-SDI, low-middle-SDI, middle-SDI, high-middle-SDI, and high-SDI quintile [15].

### Complementary Analyses

Age standardization was considered necessary when comparing rates across multiple locations or between different time periods. In GBD 2019, age-standardized rate was computed by direct standardization using the global age structure. The association between SDI and proportions of age-standardized deaths and DALYs attributable to low PA was assessed using a LOESS regression [16]. Uncertainty was propagated by sampling 1000 draws at each step of the calculation process [10, 11]. Final estimates were calculated as the mean across 1000 draws, and the 95% uncertainty intervals (UIs) were determined as the 2.5^th^ and 97.5^th^ percentiles of all 1000 draws. For all estimates, a 95% UI excluding zero was considered statistically significant.

## RESULTS

### Overall impact of low PA

In 2019, low PA potentially contributed to 0.83 million (95% UI 0.43 to 1.47) deaths and 15.75 million (95% UI 8.52 to 28.62) DALYs globally, corresponding an increase of 83.9% (95% UI 69.3 to 105.7) and 82.9% (95% UI 65.5 to 112.1) since 1990, respectively. The age-standardized rates of low-PA-related deaths and DALYs in 2019 were 11.1 (95% UI 5.7 to 19.5) per 100,000 people and 198.4 (95% UI 108.2 to 360.3) per 100,000 people, respectively (Table 1). Between 1990 and 2019, there was a decrease of -25.9% (95% UI -30.9 to -17.0) for age-standardized rate of low-PA-related deaths and a decrease of -18.3% (95% UI -25.2 to -5.5) for age-standardized rate of low-PA-related DALYs (Table 1).

**TABLE 1:** Age-standardized deaths and DALYs attributable to low physical activity in 2019 and percentage change from 1990 to 2019, overall and by sex.

	Deaths				DALYs			
	2019 agestandardized rates per 100 000 people	Percentage change in agestandardized rates, 1990–2019	2019 agestandardized PAF	Percentage change in agestandardized PAF, 1990–2019	2019 agestandardized rates per 100 000 people	Percentage change in agestandardized rates, 1990–2019	2019 agestandardized PAF	Percentage change in agestandardized PAF, 1990–2019
**Both sexes**								
All causes	11.1 (5.7 to 19.5)	-25.9% (-30.9 to -17.0)	1.5% (0.8 to 2.6)	12.2% (5.4 to 25.0)	198.4 (108.2 to 360.3)	-18.3% (-25.2 to -5.5)	0.6% (0.3 to 1.1)	24.5% (14.0 to 44.0)
Ischemic heart disease	6.5 (2.4 to 13.3)	-31.0% (-35.2 to -24.3)	5.5% (2.0 to 11.3)	-0.4% (-4.7 to 7.9)	96.4 (33.5 to 210.8)	-30.2% (-35.1 to -23.0)	4.3% (1.5 to 9.3)	-2.3% (-6.9 to 6.2)
Ischemic stroke	2.1 (0.4 to 5.3)	-32.8% (-38.3 to -23.7)	4.8% (1.0 to 12.3)	1.2% (-4.1 to 12.3)	31.2 (5.7 to 82.0)	-28.4% (-34.3 to -19.7)	3.9% (0.7 to 10.4)	0.0% (-5.9 to 9.8)
Type 2 diabetes mellitus	1.6 (0.8 to 2.7)	9.3% (2.6 to 17.3)	8.8% (4.4 to 14.5)	-1.4% (-4.6 to 3.8)	55.9 (27.2 to 97.6)	24.3% (16.8 to 32.3)	7.0% (3.4 to 12.0)	-2.6% (-6.2 to 2.5)
Colon and rectum cancer	0.8 (0.2 to 1.5)	-6.7% (-13.1 to 3.2)	5.6% (1.6 to 10.7)	-2.4% (-7.1 to 6.6)	12.6 (3.4 to 24.2)	-7.5% (-13.9 to 2.3)	4.3% (1.1 to 8.3)	-3.3% (-8.5 to 5.8)
Breast cancer	0.1 (0.1 to 0.2)	-13.7% (-20.9 to -3.9)	1.2% (0.6 to 2.1)	-1.8% (-7.0 to 7.3)	2.4 (1.2 to 4.2)	-12.7% (-20.4 to -2.3)	1.0% (0.5 to 1.7)	-2.9% (-8.5 to 6.4)

**Male**								
All causes	11.3 (5.5 to 21.3)	-22.1% (-28.3 to -9.9)	1.3% (0.6 to 2.4)	16.3% (7.8 to 34.3)	205.5 (102.9 to 388.8)	-15.3% (-23.6 to 2.6)	0.6% (0.3 to 1.1)	28.6% (16.4 to 54.7)
Ischemic heart disease	6.9 (2.3 to 15.2)	-28.4% (-33.6 to -18.7)	4.8% (1.6 to 10.4)	1.5% (-4.0 to 14.6)	108.0 (34.5 to 251.4)	-27.8% (-33.6 to -17.8)	3.7% (1.2 to 8.5)	-0.4% (-6.9 to 13.1)
Ischemic stroke	2.0 (0.3 to 5.5)	-27.6% (-35.2 to -13.9)	4.1% (0.6 to 11.4)	1.7% (-5.0 to 17.6)	29.9 (4.5 to 85.1)	-24.4% (-32.3 to -10.9)	3.4% (0.5 to 9.7)	0.8% (-6.5 to 14.8)
Type 2 diabetes mellitus	1.6 (0.8 to 2.7)	19.8% (11.1 to 30.6)	8.1% (4.0 to 13.9)	2.7% (-1.7 to 10.7)	54.3 (25.0 to 97.6)	34.2% (25.6 to 45.2)	6.3% (2.9 to 11.1)	1.1% (-3.5 to 8.5)
Colon and rectum cancer	0.8 (0.2 to 1.6)	3.3% (-6.5 to 19.9)	4.9% (1.2 to 9.7)	0.5% (-6.3 to 14.9)	13.3 (3.1 to 26.4)	2.9% (-6.9 to 19.0)	3.7% (0.9 to 7.3)	-0.7% (-7.9 to 14.2)
Breast cancer	—	—	—	—	—	—	—	—

**Female**								
All causes	10.8 (5.9 to 17.7)	-28.2% (-33.6 to -19.6)	1.8% (1.0 to 2.9)	11.8% (4.2 to 24.0)	190.9 (109.4 to 324.6)	-20.5% (-27.7 to -9.7)	0.6% (0.4 to 1.1)	22.0% (11.5 to 38.0)
Ischemic heart disease	6.1 (2.4 to 12.0)	-32.8% (-37.6 to -25.3)	6.5% (2.5 to 12.5)	0.1% (-4.5 to 9.5)	85.2 (32.0 to 174.9)	-32.5% (-37.9 to -24.8)	5.2% (1.9 to 10.4)	-2.6% (-7.0 to 5.5)
Ischemic stroke	2.1 (0.4 to 5.2)	-35.2% (-41.1 to -24.4)	5.4% (1.2 to 13.1)	4.0% (-1.0 to 16.1)	31.7 (6.5 to 79.7)	-30.4% (-36.4 to -20.7)	4.4% (0.9 to 11.2)	1.8% (-4.0 to 11.5)
Type 2 diabetes mellitus	1.6 (0.8 to 2.6)	2.6% (-5.9 to 11.7)	9.4% (4.8 to 15.2)	-2.9% (-6.4 to 2.4)	57.5 (28.6 to 98.4)	17.2% (9.1 to 25.5)	7.7% (3.9 to 13.0)	-4.2% (-7.8 to 0.5)
Colon and rectum cancer	0.7 (0.2 to 1.3)	-13.6% (-20.1 to -4.3)	6.5% (2.0 to 11.9)	-0.8% (-5.2 to 7.9)	12.0 (3.6 to 22.6)	-15.4% (-22.1 to -5.7)	5.1% (1.4 to 9.4)	-1.6% (-6.6 to 7.5)
Breast cancer	0.2 (0.1 to 0.3)	-11.0% (-18.8 to -0.9)	1.2% (0.6 to 2.1)	-0.5% (-5.8 to 8.2)	4.6 (2.3 to 8.0)	-11.2% (-19.3 to -0.6)	1.0% (0.5 to 1.7)	-1.6% (-7.4 to 7.6)

Abbreviations: DALYs = disability-adjusted life years; PAF = population attributable fraction.

By sex, in 2019, the age-standardized rates of low-PA-related deaths and DALYs were similar between males and females (Table 1). Across all age groups, the rates of low-PA-related deaths and DALYs tended to rise with increasing age in both sexes (Fig. S1). However, due to declining size of the global population with increasing age, the numbers of low-PA-related deaths and DALYs peaked at the age group of 80–84 years in both sexes (Fig. S1).

Across the 21 GBD regions, the highest 2019 age-standardized rates of low-PA-related deaths [34.8 (95% UI 20.4 to 54.4) per 100,000 people] and DALYs [671.9 (95% UI 389.1 to 1089.5) per 100,000 people] were both seen in North Africa and Middle East (Table S1). While the lowest age-standardized rates of low-PA-related deaths and DALYs were observed in Southern Latin America [3.2 (95% UI 1.6 to 6.3) per 100,000 people] and Eastern Sub-Saharan Africa [61.3 (95% UI 27.9 to 132.7) per 100,000 people], respectively (Table S1). Between 1990 and 2019, the largest percentage increase in age-standardized rates of low-PA-related deaths and DALYs was seen in Southern Sub-Saharan Africa [35.3% (95% UI 22.1 to 51.0)] and Oceania [33.1% (95% UI 11.4 to 61.7)], respectively (Table S1). In contrast, high-income Asia Pacific had the largest percentage decreases in age-standardized rates of low-PA-related deaths [-57.8% (95% UI -64.6 to -46.6)] and DALYs [-45.0% (95% UI -54.1 to -28.4)] (Table S1).

**FIG. 1:**
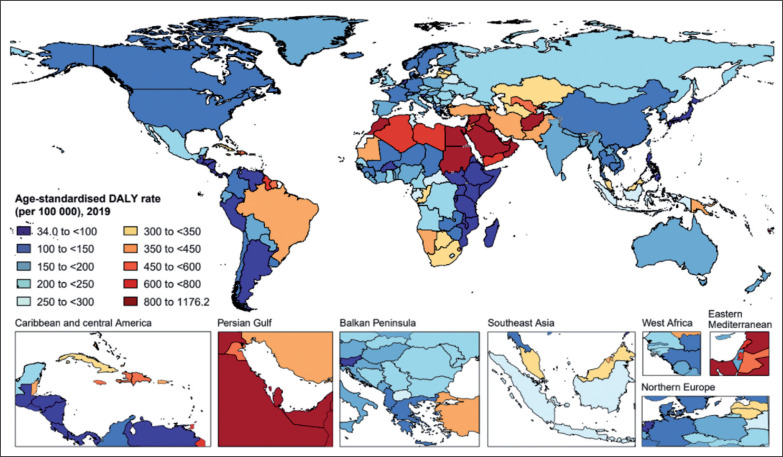
Map of age-standardized DALY rate attributable to low physical activity for both sexes combined in 2019. DALY = disability-adjusted life year.

**FIG. 2:**
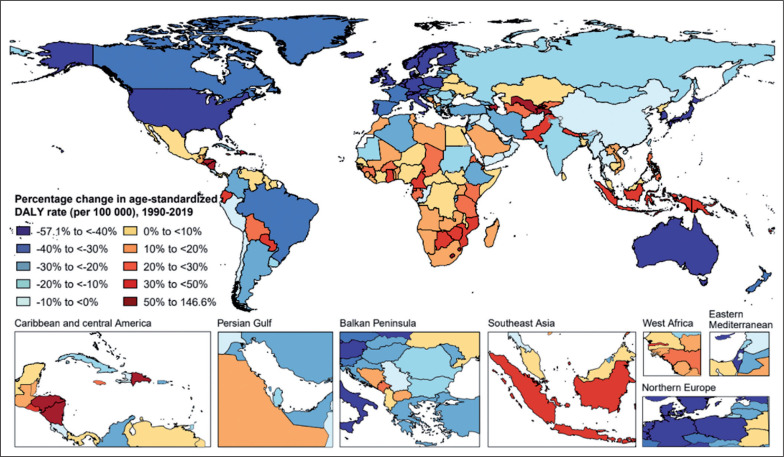
Map of percentage change in age-standardized DALY rate attributable to low physical activity for both sexes combined, 1990–2019. DALY = disability-adjusted life year.

In 2019, the age-standardized rates of low-PA-related deaths and DALYs varied widely across all countries and territories (Fig. 1 and Fig. S2). The highest age-standardized rates of low-PA-related deaths and DALYs were seen in Qatar [64.5 (95% UI 41.3 to 91.2) per 100,000 people] and Sudan [1176.2 (95% UI 725.6 to 1775.0) per 100,000 people], respectively (Fig. 1, Fig. S2, and Table S1). While Argentina was the country with the lowest age-standardized rates of low-PA-related deaths [1.9 (95% UI 0.9 to 4.3) per 100,000 people] and DALYs [34.0 (95% UI 16.1 to 74.9) per 100,000 people]. Between 1990 and 2019, Uzbekistan had the largest percentage increase in age-standardized rates of low-PA-related deaths [159.0% (95% UI 118.8 to 207.0)] and DALYs [146.6% (95% UI 98.5 to 195.9)], while Singapore had the largest percentage decrease in age-standardized rates of low-PA-related deaths [-62.5% (95% UI -69.2 to -53.6)] and DALYs [-57.1% (95% UI -65.2 to -44.0)] (Fig. 2, Fig. S3, and Table S1).

### Impact of low PA on each disease

Globally, ischemic heart disease was the leading cause of low-PA-related DALYs [96.4 (95% UI 33.5 to 210.8) per 100,000 people] in 2019, followed by type 2 diabetes mellitus [55.9 (95% UI 27.2 to 97.6) per 100,000 people], ischemic stroke [31.2 (95% UI 5.7 to 82.0) per 100,000 people], colon and rectum cancer [12.6 (95% UI 3.4 to 24.2) per 100,000 people], and breast cancer [2.4 (95% UI 1.2 to 4.2) per 100,000 people] (Table 1). Of all ischemic heart disease age-standardized DALYs worldwide, 4.3% (95% UI 1.5 to 9.3) was attributable to low PA; the corresponding proportions were 7.0% (95% UI 3.4 to 12.0) for type 2 diabetes mellitus, 3.9% (95% UI 0.7 to 10.4) for ischemic stroke, 4.3% (95% UI 1.1 to 8.3) for colon and rectum cancer, and 1.0% (95% UI 0.5 to 1.7) for breast cancer (Table 1).

**FIG. 3:**
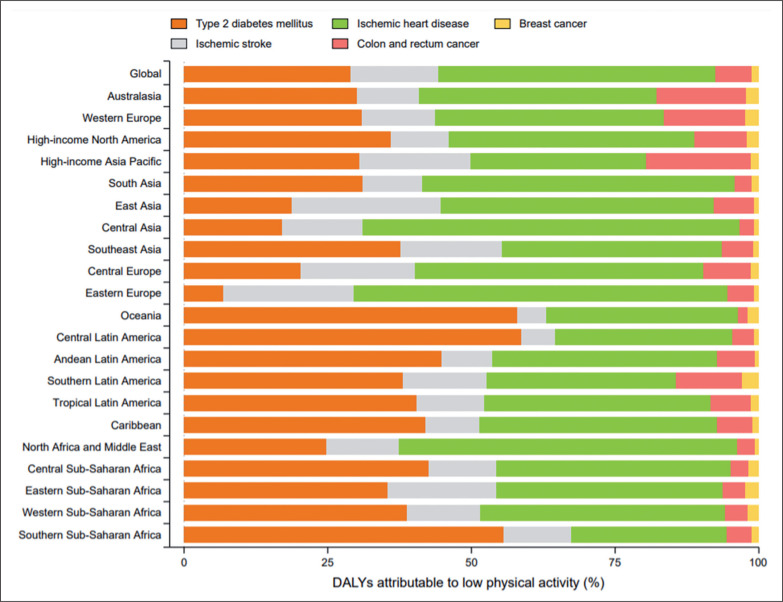
Regional variation in DALYs attributable to low physical activity in 2019, by cause. DALYs = disability-adjusted life years.

The distribution of diseases that contributed to low-PA-related DALYs varied by GBD region (Fig. 3). Ischemic heart disease was the largest contributor to low-PA-related DALYs in most regions, particularly Central Asia, Eastern Europe, and North Africa and Middle East (Fig. 3). Type 2 diabetes mellitus accounted for the largest proportion of low-PA-related DALYs in Oceania, Latin America, Caribbean, Central and Southern Sub-Saharan Africa (Fig. 3). Moreover, colon and rectum cancer accounted for a relative higher proportion of low-PA-related DALYs in high-income Asia Pacific, Australasia, and Western Europe than in other GBD regions (Fig. 3).

### Association between SDI and proportions of overall burden attributable to low PA

**FIG. 4:**
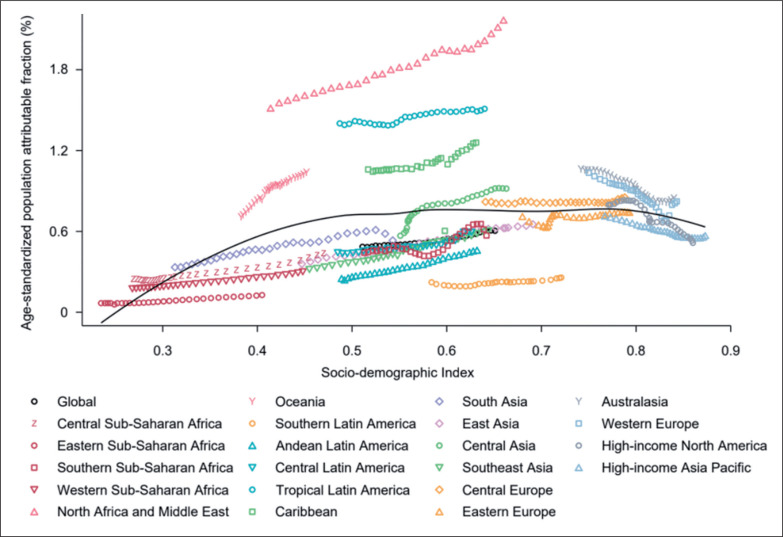
Fraction of age-standardized DALYs from all causes attributable to low physical activity across 21 GBD regions by Socio-demographic Index for both sexes combined, 1990–2019. For each region, points from left to right depict estimates from each year from 1990 to 2019. DALYs = disability-adjusted life years; GBD = Global Burden of Disease, Injuries, and Risk Factors Study.

**FIG. 5:**
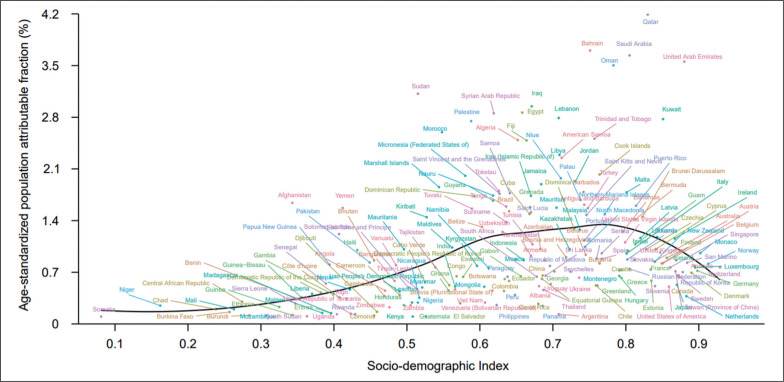
Fraction of age-standardized DALYs from all causes attributable to low physical activity across 204 countries and territories by Socio-demographic Index for both sexes combined in 2019. DALYs = disability-adjusted life years.

Of all age-standardized DALYs globally in 2019, 0.6% (95% UI 0.3 to 1.1) was attributable to low PA (Table 1). By SDI quintile, only countries in the high-SDI quintile had decreased proportions of age-standardized DALYs attributable to low PA during 1990–2019, while countries in other SDI quintiles had increased proportions in the same timeframe (Table S1). Fig. 4 depicts the changes in the attributable proportions across the 21 GBD regions by SDI from 1990 to 2019. Similarly, only the 4 regions with the highest SDI had decreased proportions of age-standardized DALYs attributable to low PA during 1990–2019, while other regions tended to have increased proportions (Fig. 4). Fig. 5 shows the association between SDI and the proportions of age-standardized DALYs attributable to low PA across 204 countries and territories in 2019. Across all countries and territories, as SDI increases, the proportions of age-standardized DALYs attributable to low PA tended to increase until SDI is about 0.77, but then decreases with higher SDI. Qatar, Bahrain, Saudi Arabia, and many other countries had much higher proportions of age-standardized DALYs attributable to low PA than expected based solely on SDI.

## DISCUSSION

Strong evidence reveals that insufficient PA presents a major global public health issue. This study aimed to provide a comprehensive estimation of the worldwide epidemiology of low-PA-generated (less than 3000-4500 MET minutes per week) disease burden. The present findings suggest that since 1990, the contribution of low PA to deaths and DALYs have increased by 83.9% and 82.9%, respectively, reaching an alarming level of 0.83 million for deaths and 15.75 million for DALYs in 2019.

These findings confirm previous reports implicating low PA as a major risk factor for decreased life expectancy [4, 5] and the development of many adverse health conditions, including cardiovascular disease (CVD) [17, 18], cancer [19], and diabetes [20]. Inversely, the beneficial impact of PA has been also confirmed with regard to the aforementioned diseases [21]. By improving major CVD risk factors (e.g., cardiorespiratory fitness) and other health outcomes, large studies have shown weekly aerobic activity of 150 min at moderate intensity can decrease CVD incidence (17%) and mortality (23%), along with reduced risk of developing ischemic heart disease (25%) [22]. A recent meta-analysis including a total of 68,416 breast cancer cases concluded being physically active reduces the risk of developing breast cancer by nearly 13% [23]. Similarly, previous largescale follow-up observational studies reported an 18% to 33% lower risk of developing diabetes in physically active people [24, 25], with average a 6% lower risk of diabetes for each 500 kcal/week energy expenditure [26].

The exact mechanisms responsible for the reduced risk of mortality and developing diseases through an active lifestyle considered to be complex and multifactorial [27]. However, it is well established that compared to sedentary lifestyle, regular PA produces a kaleidoscope of beneficial biological impacts on pathways targeting specific endocrinologic, immunologic and metabolic processes [28]. Specifically, enhanced cardiac function, higher heart rate variability, healthier blood vessels, lower amounts of visceral fat, lower blood pressure, better lipid profiles (LDL and HDL subfractions), lower markers of systemic inflammation, and improved antioxidant, immunological and endocrinological systems have been previously reported in physically active populations [27].

Current public health goals focus on reducing disease burden by extending “healthspan”, and providing extra years spent free of chronic conditions. The highest 2019 age-standardized rates of low-PA-related DALYs (per 100,000 people) seen in North Africa and Middle East (≈ 672), Oceania (≈ 512), Tropical Latin America and Caribbean (≈ 440), central Asia (≈ 317), and Southern Sub-Saharan Africa (≈300), as well as, alarming increases (1990-2019) in low-PA-related DALYs seen in most of Oceania (33.1%), central Asia (30.7%), Southern (25.7%) and Western (10.6%) Sub-Saharan Africa, and Southeast Asia (21%) countries indicates an urgent need for the development, implementation and fostering of an anti-low PA action plan in these regions. Previous reports estimate that decreasing inactivity by just 10% could prevent more than 533,000 deaths/year; this number is projected rise to over 1.3 million through a 25% reduction [6]. Therefore, disseminating appropriate PA recommendations especially in population subgroups that are most vulnerable to low-PA-related disease risk, becomes crucial.

Regarding the impact of age, the rates of low-PA-related deaths and DALYs tended to rise with increasing age in both sexes. This progressive higher impact of an inactive lifestyle on older aged adults can be explained by the gradual decline of physically active lifestyles and the gradual increase in the disease vulnerability [29]. Recent reports identify young- and middle-aged individuals as the most attractive targets for interventions to reduce the onset of noncommunicable diseases and extend healthspan [30, 31]. Therefore, to prevent DALYs in older populations, it may be advantageous to foster adherence to an active lifestyle in early age, when organs are not yet damaged.

The development status of regions and countries is one of the important factors of low-PA-generated disease burden. At the regional level, regions with the highest SDI largely decreased their proportions of age standardized DALYs attributable to low PA during 1990-2019, while other regions tended to have increased proportions in the same timeframe. This phenomenon suggests different transitions in PA and related disease spectrum across regions with various development status over the last decades. At the national level, a positive association was inversed from an SDI of about 0.77, where the age-standardized population attributable fraction (%) was highest and started to decrease. Therefore, it is recommended that a global anti-low PA action plan could be initially implemented in regions with an SDI of around 0.77.

While the results of our investigation provided a comprehensive estimation of the global disease burden attributable to low PA, it is important to point out certain limitations. First, PA data were mostly based on self-reported assessment methods (e.g., IPAQ, GPAQ questionnaires) known to be subject to certain biases [32]. However, given that PA data collected using objective methods (i.e., accelerometry) are mostly only available for high-income countries, utilizing these types of tools could make cross-country comparisons difficult. For more comparable results, a larger scale of PA data collected using objective methods could be warranted. Second, our analysis included only adult populations (aged ≥25 years) and it is well documented that PA levels can differ between different age groups [32, 33]. PA data in individuals younger than 25 years are needed to develop a more comprehensive understanding of low PA’s overall impact on the global burden of disease. Third, PA data were not available for all countries and years. However, statistically robust and sound approaches have been applied in GBD 2019 in order to overcome data scarcity in some countries and deal with uncertainty.

## CONCLUSIONS

Low PA is a an important risk factor of the considerable disease burden across the globe and seems to contribute to 0.6% of all age standardized DALYs globally in 2019. The extent of disease burden attributable to low PA varies substantially by age and across countries and is strongly associated with development status. Therefore, effective and targeted initiatives to promote PA within different age groups and countries are urgently needed. Importantly, taking into-consideration the contribution of multiple risk factors to disease burden, strategies to reduce global disease burden should not be only limited to promoting PA, but should also consider other risk factors identified in previous GBD studies [7, 11].

## Supplementary Material

Global disease burden attributed to low physical activity in 204 countries and territories from 1990 to 2019: Insights from the Global Burden of Disease 2019 Study
