# Supplementary material for: Global disease burden attributed to low physical activity in 204 countries and territories from 1990 to 2019: Insights from the Global Burden of Disease 2019 Study
Source: Biol Sport. 2022 Nov 22;40(3):835–55. doi: 10.5114/biolsport.2023.121322 (PMC10286621; doi:10.5114/biolsport.2023.121322)
Supplement: Global disease burden attributed to low physical activity in 204 countries and territories from 1990 to 2019: Insights from the Global Burden of Disease 2019 Study [file JBS-40-121322-s1.pdf]

SUPPLEMENTARY MATERIALS

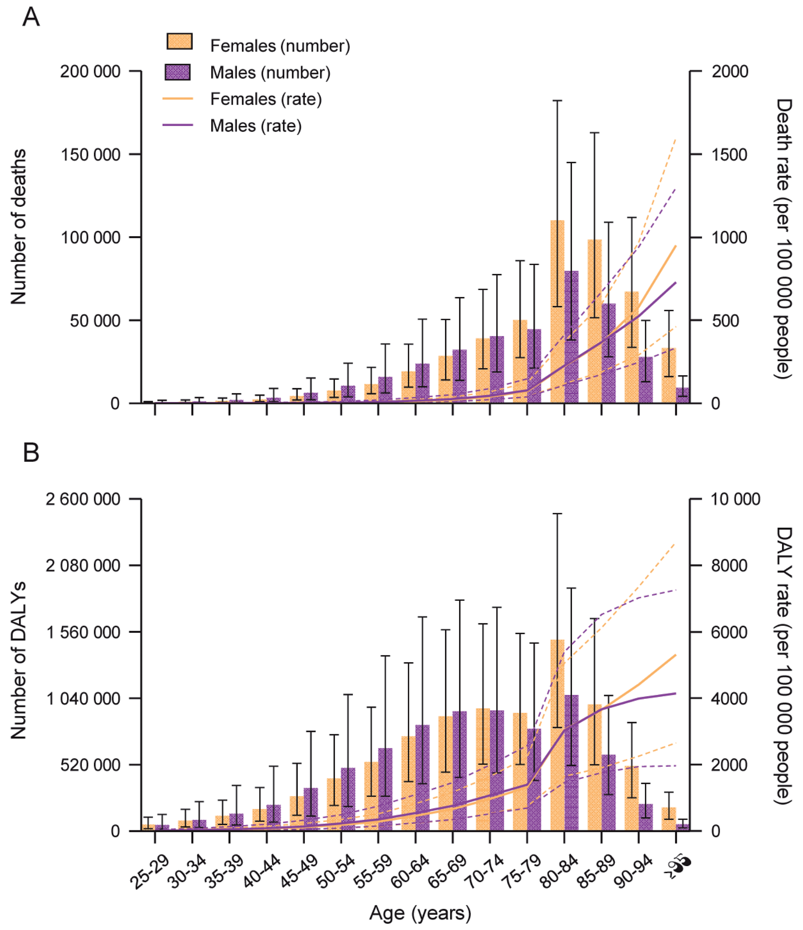

FIG. S1. Age-specific numbers and rates of deaths and DALYs attributable to low physical activity by sex, in 2019. (A) Deaths. (B) DALYs. Error bars indicate the 95% uncertainty interval. DALYs, disability-adjusted life years.

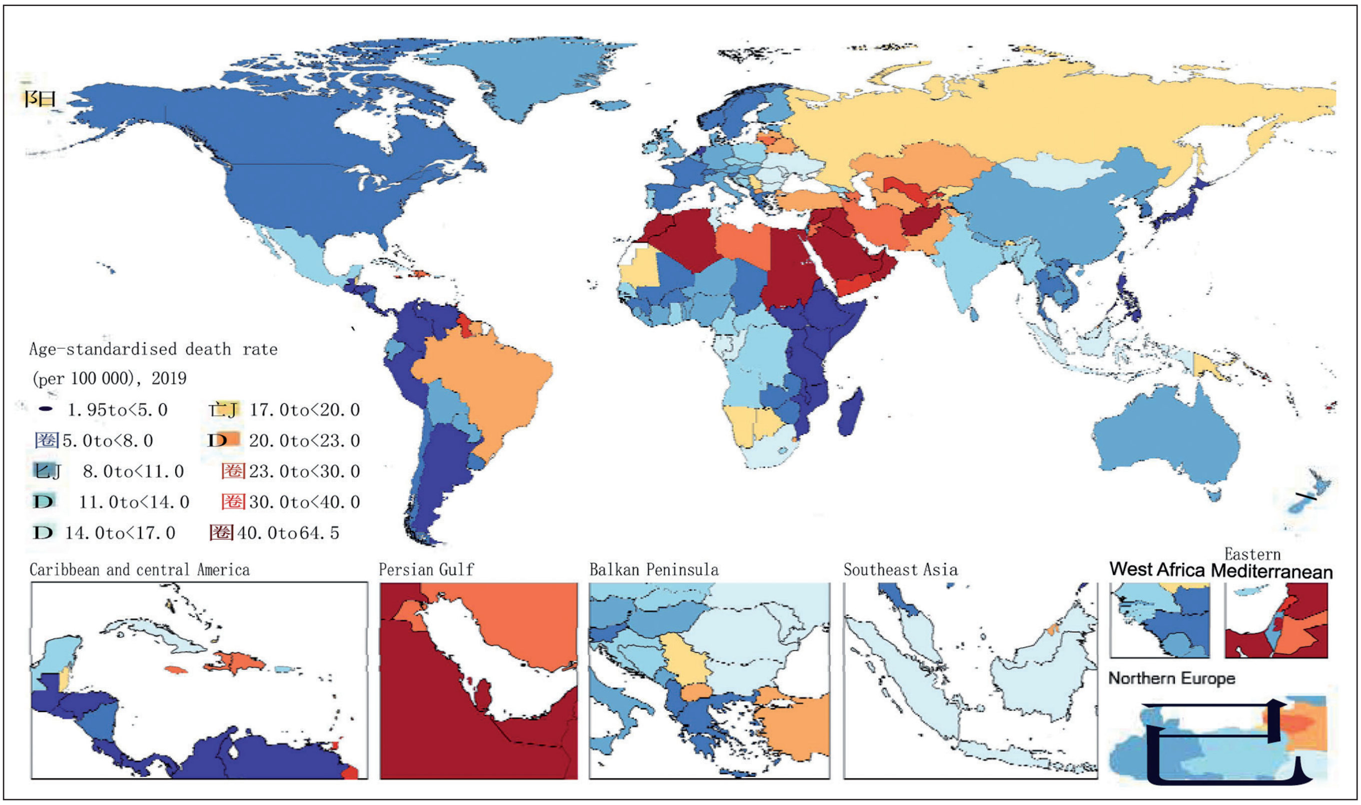

FIG. S2. Map of age-standardized death rate attributable to low physical activity for both sexes combined in 2019.

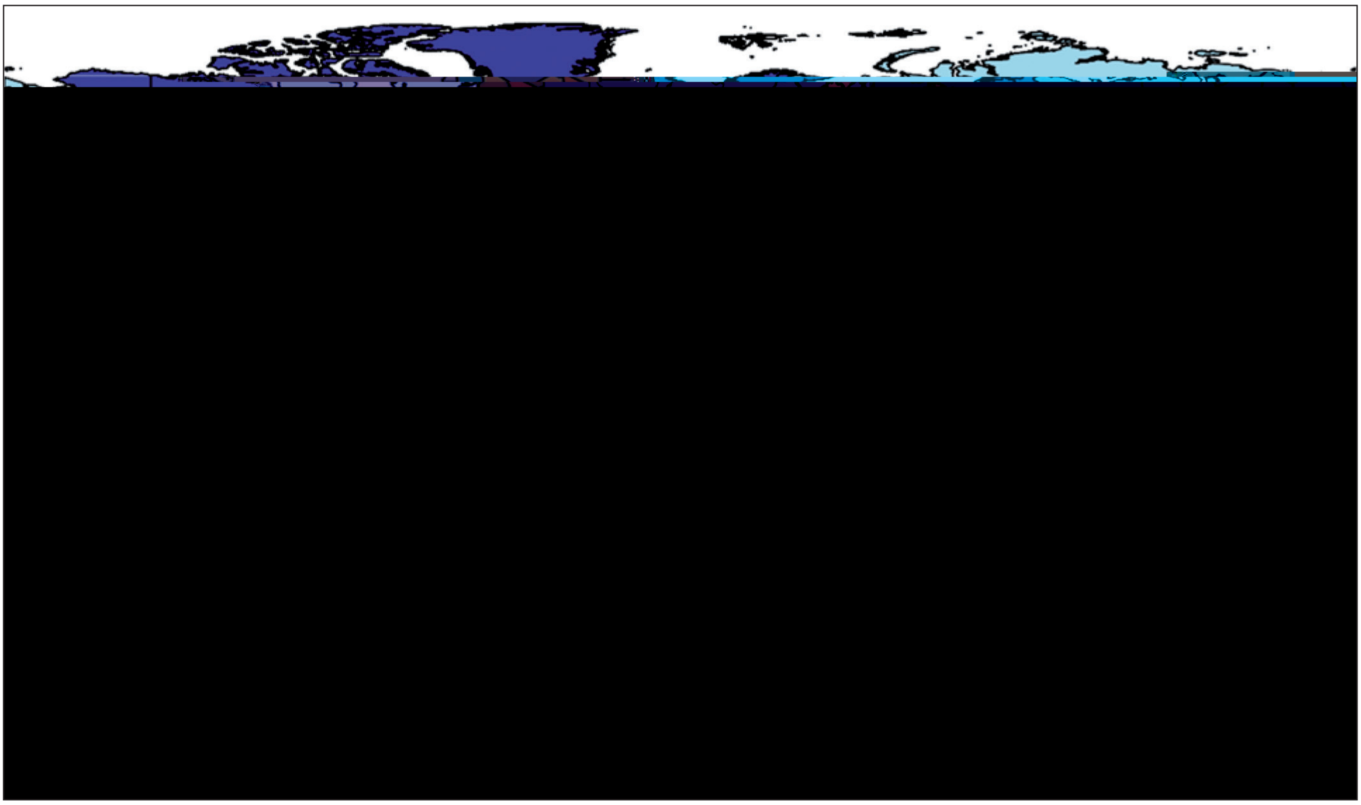

FIG. S3. Map of percentage change in age-standardized death rate attributable to low physical activity for both sexes combined, 1990–2019.

TABLE S1. Age-standardised Deaths and DALYs attributable to low physical activity for both sexes combined in 2019 and percentage change from 1990 to 2019, by SDI quintile and location.

1	2	3	4	5	6	7	8	9
	Deaths				DALYs			
	2019 age-standardised rates per 100 000 people	Percentage change in age- standardised rates, 1990–2019	2019 age-standardised PAF	Percentage change in age- standardised PAF, 1990–2019	2019 age-standardised rates per 100 000 people	Percentage change in age- standardised rates, 1990–2019	2019 age-standardised PAF	Percentage change in age- standardised PAF, 1990–2019
Global	11.1 (5.7 to 19.5)	-25.9% (-30.9 to -17.0)	1.51% (0.78 to 2.63)	12.2% (5.4 to 25.0)	198.4 (108.2 to 360.3)	-18.3% (-25.2 to -5.5)	0.60% (0.33 to 1.08)	24.5% (14.0 to 44.0)
SDI quintile								
Low-SDI quintile	10.1 (5.3 to 18.8)	-2.5% (-12.1 to 9.0)	0.88% (0.46 to 1.59)	54.0% (38.2 to 71.4)	186.9 (96.3 to 352.2)	-1.6% (-11.2 to 10.1)	0.38% (0.20 to 0.70)	68.0% (49.9 to 89.8)
Low-middle-SDI quintile	12.9 (7.0 to 22.5)	0.8% (-8.0 to 11.4)	1.36% (0.75 to 2.39)	56.4% (44.2 to 73.5)	227.5 (126.3 to 413.9)	1.0% (-8.7 to 12.0)	0.58% (0.32 to 1.02)	66.5% (51.9 to 86.9)
Middle-SDI quintile	13.1 (6.7 to 23.5)	0.4% (-9.1 to 10.6)	1.78% (0.92 to 3.14)	51.0% (38.6 to 65.4)	226.4 (118.8 to 412.4)	1.4% (-8.2 to 13.3)	0.79% (0.41 to 1.46)	57.8% (44.3 to 74.2)
High-middle-SDI quintile	12.7 (6.6 to 21.9)	-27.7% (-33.4 to -19.9)	2.09% (1.09 to 3.67)	14.2% (6.2 to 25.9)	205.7 (113.5 to 369.8)	-24.5% (-31.0 to -13.6)	0.86% (0.47 to 1.53)	15.6% (7.0 to 31.0)
High-SDI quintile	7.1 (3.6 to 12.3)	-52.0% (-56.7 to -42.6)	1.59% (0.82 to 2.76)	-27.3% (-34.5 to -13.0)	149.0 (82.3 to 265.1)	-37.2% (-46.6 to -17.8)	0.70% (0.39 to 1.23)	-19.7% (-31.1 to 4.6)
Central Sub-Saharan Africa	11.7 (5.8 to 22.1)	4.1% (-14.4 to 26.8)	0.93% (0.47 to 1.74)	55.9% (36.7 to 74.9)	224.3 (110.4 to 447.2)	4.0% (-13.1 to 25.8)	0.44% (0.22 to 0.83)	76.2% (55.2 to 99.5)
Angola	11.6 (5.8 to 22.7)	17.6% (-10.4 to 57.8)	0.97% (0.48 to 1.82)	103.5% (62.8 to 155.7)	222.3 (110.3 to 430.6)	12.4% (-12.3 to 47.5)	0.45% (0.23 to 0.85)	124.3% (84.4 to 174.4)
Central African Republic	13.2 (6.3 to 26.0)	6.9% (-16.2 to 36.9)	0.63% (0.31 to 1.18)	20.4% (6.3 to 37.9)	264.8 (127.0 to 529.6)	6.8% (-15.7 to 35.0)	0.30% (0.14 to 0.57)	27.6% (11.9 to 46.0)
Congo	16.2 (8.2 to 29.6)	1.5% (-19.1 to 28.7)	1.29% (0.67 to 2.38)	62.0% (41.1 to 93.2)	311.2 (158.4 to 595.4)	-1.0% (-21.1 to 25.1)	0.65% (0.33 to 1.20)	69.6% (46.4 to 100.0)
Democratic Republic of the Congo	11.1 (5.4 to 21.1)	0.3% (-20.2 to 25.1)	0.91% (0.45 to 1.71)	46.7% (27.4 to 67.4)	213.6 (102.3 to 419.4)	1.6% (-17.9 to 25.8)	0.43% (0.21 to 0.79)	69.1% (46.0 to 95.4)
Equatorial Guinea	15.4 (8.0 to 27.0)	39.6% (-3.4 to 113.4)	1.33% (0.71 to 2.25)	154.0% (72.7 to 269.3)	289.0 (153.2 to 517.9)	30.7% (-8.0 to 93.6)	0.61% (0.33 to 1.05)	160.5% (82.5 to 263.0)
Gabon	15.0 (7.9 to 26.5)	21.6% (-1.2 to 50.7)	1.35% (0.70 to 2.34)	65.4% (42.0 to 97.9)	276.3 (141.9 to 511.1)	21.4% (-1.9 to 50.6)	0.65% (0.33 to 1.17)	76.9% (50.1 to 111.5)
Eastern Sub-Saharan Africa	3.4 (1.6 to 7.0)	4.2% (-14.9 to 22.7)	0.29% (0.14 to 0.60)	76.4% (44.2 to 104.8)	61.3 (27.9 to 132.7)	-0.1% (-16.3 to 15.2)	0.13% (0.06 to 0.27)	89.3% (57.9 to 118.6)
Burundi	3.4 (1.5 to 7.7)	-10.4% (-33.1 to 16.7)	0.27% (0.11 to 0.60)	56.8% (27.0 to 95.3)	61.5 (27.2 to 146.8)	-13.9% (-34.8 to 10.8)	0.12% (0.05 to 0.27)	63.6% (33.1 to 101.4)
Comoros	2.7 (1.1 to 6.6)	3.6% (-18.8 to 41.5)	0.27% (0.11 to 0.67)	60.8% (29.9 to 106.6)	46.9 (20.4 to 114.4)	1.2% (-19.4 to 47.6)	0.11% (0.05 to 0.28)	69.4% (38.2 to 144.4)
Djibouti	4.2 (1.8 to 9.1)	30.4% (1.8 to 68.1)	0.38% (0.17 to 0.80)	63.0% (33.2 to 107.4)	73.4 (32.4 to 163.8)	28.7% (2.5 to 65.9)	0.16% (0.07 to 0.35)	74.8% (45.1 to 114.3)
Eritrea	4.2 (1.8 to 9.1)	34.7% (-4.7 to 91.8)	0.31% (0.14 to 0.64)	145.9% (80.5 to 240.8)	75.0 (32.8 to 166.1)	22.8% (-7.7 to 64.8)	0.14% (0.07 to 0.32)	172.7% (110.2 to 256.2)
Ethiopia	2.8 (1.2 to 5.9)	-16.4% (-44.4 to 14.0)	0.28% (0.12 to 0.59)	100.5% (34.3 to 164.5)	49.0 (21.8 to 106.3)	-28.7% (-49.4 to -4.2)	0.12% (0.05 to 0.26)	86.6% (33.3 to 146.6)
Kenya	2.5 (1.1 to 5.7)	23.5% (5.7 to 47.7)	0.22% (0.10 to 0.49)	41.6% (22.9 to 64.9)	45.0 (19.9 to 100.5)	26.3% (9.2 to 45.6)	0.10% (0.04 to 0.22)	62.2% (41.3 to 85.7)
Madagascar	4.0 (1.6 to 9.2)	11.5% (-14.5 to 43.5)	0.33% (0.14 to 0.75)	51.6% (27.9 to 77.8)	70.0 (29.9 to 169.4)	11.0% (-12.7 to 39.9)	0.15% (0.06 to 0.35)	77.0% (51.0 to 106.0)
Malawi	4.1 (1.8 to 8.4)	6.1% (-15.2 to 33.9)	0.34% (0.16 to 0.69)	77.4% (44.9 to 119.8)	74.1 (33.8 to 153.8)	4.1% (-15.4 to 29.2)	0.15% (0.07 to 0.30)	104.1% (67.5 to 152.2)
Mozambique	4.1 (1.8 to 9.1)	34.1% (2.1 to 75.2)	0.26% (0.11 to 0.57)	59.5% (29.0 to 93.6)	75.6 (33.0 to 171.9)	37.0% (8.9 to 74.0)	0.11% (0.05 to 0.25)	90.4% (57.8 to 130.3)

TABLE S1. Continue.

1	2	3	4	5	6	7	8	9
Rwanda	3.2 (1.4 to 6.8)	-13.6% (-35.3 to 12.3)	0.32% (0.14 to 0.66)	84.9% (39.6 to 133.8)	54.7 (24.7 to 112.5)	-20.2% (-38.9 to 1.3)	0.13% (0.06 to 0.28)	85.4% (41.8 to 131.6)
Somalia	3.6 (1.6 to 8.4)	6.8% (-18.3 to 37.1)	0.22% (0.10 to 0.50)	48.4% (19.9 to 83.8)	67.1 (28.4 to 160.0)	4.1% (-19.5 to 34.0)	0.10% (0.04 to 0.24)	61.0% (31.9 to 93.0)
South Sudan	3.2 (1.4 to 7.0)	3.2% (-20.1 to 34.0)	0.27% (0.12 to 0.57)	43.2% (16.8 to 74.5)	58.0 (25.6 to 126.6)	4.8% (-18.0 to 34.1)	0.10% (0.05 to 0.22)	55.3% (26.8 to 91.8)
United Republic of Tanzania	3.6 (1.6 to 7.9)	22.9% (-1.7 to 51.2)	0.34% (0.15 to 0.74)	92.0% (56.0 to 134.4)	62.3 (27.6 to 135.1)	23.2% (0.7 to 48.7)	0.14% (0.06 to 0.30)	117.7% (81.2 to 158.6)
Uganda	3.4 (1.5 to 7.0)	14.7% (-7.5 to 45.1)	0.30% (0.14 to 0.62)	118.5% (77.9 to 172.4)	62.0 (28.3 to 129.2)	16.7% (-4.4 to 45.0)	0.13% (0.06 to 0.27)	154.9% (112.6 to 212.1)
Zambia	7.3 (3.4 to 14.5)	16.8% (-9.9 to 51.5)	0.55% (0.26 to 1.05)	72.1% (39.8 to 115.1)	136.5 (64.7 to 273.8)	14.0% (-11.3 to 44.7)	0.25% (0.12 to 0.50)	90.9% (55.3 to 138.3)
Southern Sub-Saharan Africa	15.8 (8.7 to 26.1)	35.3% (22.1 to 51.0)	1.30% (0.70 to 2.10)	28.6% (16.1 to 43.1)	300.3 (161.1 to 516.9)	25.7% (13.2 to 40.7)	0.57% (0.30 to 0.97)	29.4% (15.4 to 45.5)
Botswana	17.9 (9.0 to 32.9)	43.4% (9.4 to 94.9)	1.27% (0.66 to 2.33)	48.6% (24.2 to 80.0)	341.7 (170.9 to 653.7)	48.6% (12.7 to 99.6)	0.61% (0.31 to 1.13)	54.5% (26.9 to 90.8)
Lesotho	13.8 (6.6 to 25.8)	100.9% (52.5 to 168.3)	0.62% (0.30 to 1.12)	28.7% (6.5 to 57.8)	263.3 (123.2 to 513.5)	104.8% (55.6 to 172.2)	0.29% (0.13 to 0.56)	38.1% (13.5 to 69.5)
Namibia	18.7 (10.1 to 32.6)	13.0% (-8.3 to 38.8)	1.53% (0.83 to 2.62)	29.6% (12.9 to 51.7)	366.8 (199.6 to 655.5)	12.7% (-8.6 to 41.9)	0.76% (0.41 to 1.31)	30.4% (14.7 to 54.4)
South Africa	16.7 (9.3 to 26.5)	31.7% (18.4 to 47.5)	1.47% (0.80 to 2.35)	27.3% (14.8 to 42.2)	319.7 (173.2 to 540.5)	19.5% (6.4 to 35.3)	0.64% (0.34 to 1.08)	28.4% (13.2 to 46.6)
Eswatini	21.9 (11.2 to 39.7)	55.2% (15.7 to 106.0)	1.30% (0.68 to 2.24)	32.1% (11.8 to 57.3)	433.1 (216.4 to 799.8)	60.4% (21.8 to 114.2)	0.64% (0.32 to 1.15)	35.4% (13.3 to 61.4)
Zimbabwe	7.2 (3.2 to 15.2)	36.6% (7.0 to 74.3)	0.48% (0.22 to 1.01)	22.3% (2.0 to 52.4)	133.5 (60.8 to 277.4)	45.4% (16.9 to 79.9)	0.23% (0.10 to 0.46)	37.1% (13.8 to 72.5)
Western Sub-Saharan Africa	9.2 (4.5 to 17.5)	8.1% (-9.5 to 26.9)	0.77% (0.38 to 1.45)	51.3% (26.8 to 73.5)	165.0 (79.6 to 311.3)	10.6% (-8.1 to 30.3)	0.31% (0.15 to 0.57)	69.7% (42.3 to 97.9)
Benin	9.2 (4.4 to 18.5)	13.9% (-5.4 to 41.0)	0.78% (0.37 to 1.52)	55.2% (32.1 to 87.5)	172.5 (81.8 to 342.8)	19.4% (-3.5 to 48.8)	0.34% (0.16 to 0.64)	78.2% (49.0 to 117.8)
Burkina Faso	5.2 (2.4 to 11.2)	27.5% (5.8 to 56.6)	0.40% (0.18 to 0.83)	82.9% (53.5 to 125.4)	95.0 (43.0 to 204.5)	28.8% (6.8 to 59.7)	0.16% (0.07 to 0.34)	104.9% (70.1 to 149.6)
Cameroon	11.3 (5.7 to 21.5)	31.1% (6.2 to 65.5)	0.89% (0.44 to 1.67)	56.1% (33.1 to 88.2)	210.7 (103.5 to 402.0)	39.1% (11.0 to 75.8)	0.39% (0.19 to 0.74)	78.8% (50.7 to 115.6)
Cabo Verde	9.7 (4.8 to 19.4)	70.1% (37.0 to 136.6)	1.21% (0.59 to 2.41)	78.3% (44.3 to 149.5)	169.2 (81.9 to 325.4)	71.1% (38.9 to 125.3)	0.57% (0.27 to 1.10)	130.3% (86.7 to 201.7)
Chad	6.7 (3.0 to 14.0)	16.6% (-3.2 to 44.4)	0.48% (0.22 to 1.00)	44.7% (24.4 to 72.3)	125.0 (57.2 to 259.7)	21.8% (0.6 to 51.0)	0.20% (0.09 to 0.42)	65.8% (39.9 to 98.6)
Cote d'Ivoire	9.9 (4.7 to 19.5)	2.2% (-14.7 to 28.1)	0.81% (0.38 to 1.58)	40.5% (20.4 to 71.9)	184.9 (87.3 to 365.7)	7.0% (-13.4 to 35.9)	0.36% (0.17 to 0.71)	59.0% (33.9 to 99.7)
Gambia	10.2 (4.7 to 20.3)	32.4% (5.9 to 70.9)	0.87% (0.41 to 1.69)	61.5% (36.7 to 97.6)	179.7 (81.0 to 366.4)	36.3% (8.1 to 76.1)	0.40% (0.18 to 0.82)	91.6% (56.3 to 138.2)
Ghana	11.5 (5.7 to 22.1)	34.6% (9.7 to 69.1)	1.01% (0.49 to 1.92)	75.1% (50.1 to 108.6)	208.0 (102.1 to 392.9)	39.2% (11.7 to 77.4)	0.45% (0.22 to 0.85)	98.4% (65.6 to 143.0)
Guinea	7.5 (3.5 to 15.5)	24.0% (-1.3 to 56.5)	0.55% (0.26 to 1.09)	60.2% (36.6 to 91.6)	136.1 (63.0 to 281.8)	28.2% (2.7 to 61.6)	0.23% (0.11 to 0.46)	91.9% (64.6 to 128.0)
Guinea-Bissau	11.3 (5.2 to 23.0)	15.0% (-9.7 to 48.5)	0.76% (0.36 to 1.49)	70.6% (42.2 to 108.8)	214.1 (97.6 to 437.9)	13.5% (-10.1 to 47.4)	0.36% (0.17 to 0.72)	92.3% (61.5 to 132.8)
Liberia	10.4 (4.9 to 20.5)	4.8% (-15.9 to 39.0)	0.92% (0.45 to 1.77)	91.4% (63.7 to 131.4)	197.8 (94.6 to 375.0)	10.6% (-10.9 to 43.6)	0.41% (0.19 to 0.79)	151.6% (105.7 to 214.3)
Mali	6.8 (3.1 to 13.7)	7.2% (-12.5 to 31.8)	0.53% (0.24 to 1.07)	62.5% (33.5 to 97.0)	117.4 (53.4 to 237.6)	10.4% (-9.8 to 37.0)	0.20% (0.09 to 0.39)	82.8% (52.7 to 120.8)
Mauritania	19.8 (11.3 to 31.1)	-16.4% (-30.8 to 2.2)	2.20% (1.26 to 3.44)	41.9% (20.0 to 68.3)	368.9 (210.8 to 593.0)	-18.6% (-34.3 to 1.9)	1.01% (0.58 to 1.58)	44.1% (20.5 to 73.5)

TABLE S1. Continue.

1	2	3	4	5	6	7	8	9
Niger	8.0 (3.7 to 16.7)	3.8% (-16.3 to 30.4)	0.64% (0.29 to 1.27)	66.6% (36.5 to 105.4)	140.2 (64.0 to 286.1)	6.4% (-13.4 to 33.0)	0.25% (0.11 to 0.49)	99.8% (66.3 to 147.8)
Nigeria	8.8 (4.3 to 16.9)	-0.3% (-25.2 to 27.0)	0.77% (0.38 to 1.47)	44.0% (11.3 to 70.5)	154.1 (72.9 to 296.6)	0.3% (-23.9 to 28.1)	0.29% (0.14 to 0.56)	57.0% (23.5 to 92.1)
Sao Tome and Principe	9.9 (4.5 to 20.0)	41.0% (16.7 to 71.5)	1.00% (0.45 to 1.97)	76.3% (47.9 to 114.8)	172.7 (81.4 to 344.9)	43.2% (18.9 to 73.0)	0.49% (0.23 to 0.98)	121.2% (86.8 to 167.2)
Senegal	11.5 (5.8 to 21.5)	4.9% (-11.6 to 30.0)	1.13% (0.57 to 2.15)	54.4% (30.9 to 87.9)	227.9 (114.1 to 433.5)	8.0% (-11.6 to 35.5)	0.55% (0.27 to 1.01)	76.8% (45.8 to 119.3)
Sierra Leone	9.3 (4.2 to 19.1)	9.2% (-10.9 to 41.8)	0.71% (0.33 to 1.43)	54.7% (32.2 to 89.0)	163.2 (74.2 to 328.8)	13.6% (-8.7 to 48.6)	0.28% (0.13 to 0.55)	82.5% (52.7 to 125.9)
Togo	9.5 (4.4 to 19.3)	10.1% (-8.3 to 37.8)	0.80% (0.38 to 1.55)	41.1% (23.0 to 67.3)	169.0 (75.9 to 346.9)	14.0% (-5.5 to 43.6)	0.34% (0.16 to 0.68)	60.3% (38.4 to 93.4)
Andean Latin America	6.1 (2.7 to 12.0)	-2.5% (-22.5 to 38.6)	1.05% (0.49 to 2.00)	56.8% (29.6 to 114.0)	116.0 (53.7 to 223.2)	8.9% (-15.0 to 53.0)	0.45% (0.21 to 0.84)	86.5% (50.0 to 153.0)
Bolivia (Plurinational State of)	9.0 (3.9 to 18.8)	19.7% (-8.9 to 65.9)	1.00% (0.44 to 1.97)	77.7% (44.3 to 132.9)	157.5 (68.8 to 314.7)	20.3% (-8.9 to 65.5)	0.47% (0.21 to 0.91)	112.1% (69.2 to 179.1)
Ecuador	8.4 (3.9 to 15.9)	25.6% (-1.5 to 77.7)	1.28% (0.61 to 2.48)	53.8% (28.9 to 108.9)	155.5 (73.1 to 295.4)	34.2% (4.9 to 83.0)	0.58% (0.27 to 1.07)	79.3% (46.8 to 137.2)
Peru	4.5 (2.0 to 8.9)	-20.5% (-42.5 to 18.0)	0.98% (0.44 to 1.87)	49.2% (18.3 to 110.2)	88.8 (40.1 to 169.8)	-7.8% (-33.0 to 38.5)	0.39% (0.19 to 0.73)	74.9% (34.8 to 154.0)
Tropical Latin America	20.2 (13.3 to 28.3)	-37.5% (-44.8 to -23.3)	3.19% (2.11 to 4.47)	-0.4% (-12.2 to 22.2)	442.2 (282.8 to 640.5)	-30.6% (-38.8 to -15.5)	1.51% (0.98 to 2.18)	7.7% (-5.7 to 31.0)
Brazil	20.4 (13.5 to 28.6)	-38.4% (-45.5 to -24.2)	3.23% (2.13 to 4.50)	-0.7% (-12.4 to 21.7)	448.6 (286.9 to 645.8)	-31.1% (-39.4 to -16.1)	1.53% (0.99 to 2.20)	7.7% (-5.8 to 30.8)
Paraguay	9.5 (4.3 to 19.1)	31.8% (-3.6 to 90.8)	1.54% (0.70 to 2.99)	33.6% (7.5 to 87.0)	172.6 (74.0 to 346.3)	49.9% (8.5 to 110.8)	0.64% (0.28 to 1.25)	64.7% (27.3 to 123.0)
Central Latin America	7.7 (3.6 to 15.5)	-5.7% (-18.3 to 10.0)	1.24% (0.58 to 2.42)	30.1% (14.4 to 51.4)	164.4 (75.5 to 318.2)	-3.1% (-15.4 to 10.6)	0.60% (0.27 to 1.13)	33.7% (18.2 to 52.1)
Colombia	4.7 (1.9 to 10.2)	-32.6% (-48.1 to -10.6)	1.01% (0.42 to 2.13)	18.5% (-2.1 to 49.9)	103.5 (44.5 to 210.5)	-20.8% (-39.4 to 4.9)	0.45% (0.20 to 0.88)	23.5% (-3.1 to 61.2)
Costa Rica	3.0 (1.2 to 6.9)	-18.1% (-38.7 to 13.9)	0.62% (0.24 to 1.43)	3.4% (-16.8 to 35.8)	60.2 (25.4 to 128.7)	-6.5% (-28.1 to 25.4)	0.27% (0.12 to 0.58)	9.4% (-13.0 to 42.6)
El Salvador	4.2 (1.6 to 9.8)	35.7% (-7.2 to 102.0)	0.65% (0.26 to 1.49)	76.4% (26.8 to 151.9)	80.3 (32.1 to 176.9)	41.6% (-6.0 to 106.4)	0.28% (0.11 to 0.61)	108.5% (42.4 to 192.9)
Guatemala	2.0 (0.8 to 5.2)	17.3% (-18.5 to 97.5)	0.24% (0.10 to 0.62)	102.1% (39.6 to 227.6)	35.5 (15.3 to 90.2)	15.3% (-25.4 to 98.8)	0.10% (0.05 to 0.26)	104.5% (31.7 to 240.7)
Honduras	4.8 (1.7 to 11.7)	75.3% (36.9 to 132.7)	0.54% (0.19 to 1.32)	72.6% (35.6 to 127.6)	82.7 (32.2 to 188.4)	53.7% (18.6 to 96.5)	0.25% (0.10 to 0.58)	98.3% (56.8 to 151.1)
Mexico	11.4 (5.4 to 21.3)	1.6% (-12.7 to 19.7)	1.73% (0.83 to 3.28)	33.9% (19.4 to 52.6)	245.1 (113.7 to 461.3)	0.8% (-11.6 to 15.6)	0.87% (0.40 to 1.61)	34.1% (21.4 to 50.6)
Nicaragua	6.0 (2.2 to 14.1)	88.9% (37.1 to 147.1)	0.83% (0.31 to 1.99)	87.6% (36.4 to 144.7)	96.0 (38.2 to 217.5)	63.1% (20.0 to 107.1)	0.35% (0.14 to 0.78)	122.8% (63.5 to 179.2)
Panama	3.0 (1.2 to 6.7)	-6.1% (-32.8 to 39.6)	0.63% (0.25 to 1.39)	20.1% (-9.1 to 72.6)	56.9 (23.5 to 117.5)	5.3% (-26.4 to 50.3)	0.24% (0.10 to 0.49)	26.8% (-9.1 to 75.7)
Venezuela (Bolivarian Republic of)	4.6 (1.7 to 11.3)	1.6% (-22.7 to 37.9)	0.68% (0.26 to 1.61)	18.4% (-4.1 to 48.5)	84.9 (34.1 to 198.7)	3.0% (-20.6 to 35.9)	0.29% (0.12 to 0.66)	18.3% (-3.6 to 45.5)
Southern Latin America	3.2 (1.6 to 6.3)	-26.1% (-42.3 to 5.5)	0.53% (0.26 to 1.04)	1.0% (-21.2 to 46.0)	62.8 (32.7 to 121.3)	-11.9% (-35.6 to 29.8)	0.26% (0.13 to 0.49)	15.6% (-15.2 to 71.5)
Argentina	1.9 (0.9 to 4.3)	-30.9% (-47.9 to 7.1)	0.30% (0.14 to 0.67)	-11.7% (-33.4 to 36.8)	34.0 (16.1 to 74.9)	-25.4% (-45.4 to 13.5)	0.13% (0.06 to 0.29)	-5.0% (-30.6 to 46.1)
Chile	5.2 (2.6 to 10.1)	-30.6% (-46.5 to 5.0)	1.07% (0.53 to 2.02)	13.8% (-12.3 to 72.7)	112.1 (57.4 to 205.4)	-9.5% (-33.3 to 39.6)	0.52% (0.27 to 0.93)	29.9% (-3.4 to 100.6)
Uruguay	6.8 (2.8 to 13.5)	-25.3% (-40.4 to 2.9)	1.13% (0.48 to 2.24)	0.1% (-19.4 to 37.7)	132.4 (58.8 to 262.6)	-14.3% (-33.6 to 25.4)	0.54% (0.24 to 1.05)	8.8% (-15.5 to 60.2)

TABLE S1. Continue.

1	2	3	4	5	6	7	8	9
Caribbean	20.7 (11.6 to 33.0)	-17.6% (-29.0 to -2.9)	2.73% (1.54 to 4.27)	3.2% (-5.1 to 16.5)	438.9 (247.0 to 726.3)	-4.1% (-17.4 to 12.5)	1.26% (0.72 to 2.04)	18.8% (7.7 to 35.0)
Antigua and Barbuda	21.4 (12.5 to 32.9)	-0.6% (-14.8 to 18.9)	3.13% (1.83 to 4.70)	11.0% (-2.1 to 29.9)	415.0 (238.2 to 656.9)	5.3% (-10.4 to 26.0)	1.61% (0.92 to 2.54)	19.1% (3.2 to 40.7)
Bahamas	17.8 (10.1 to 28.3)	-19.8% (-32.9 to -2.2)	2.35% (1.35 to 3.64)	-5.8% (-14.1 to 6.8)	392.6 (223.3 to 653.0)	-8.9% (-23.6 to 10.2)	1.25% (0.72 to 2.03)	2.0% (-8.3 to 17.4)
Barbados	23.4 (14.3 to 34.5)	-19.9% (-32.9 to -3.6)	3.49% (2.21 to 5.04)	-7.3% (-17.1 to 8.0)	465.8 (282.6 to 713.9)	-9.4% (-23.5 to 7.9)	1.79% (1.10 to 2.70)	3.0% (-7.7 to 18.8)
Belize	18.4 (10.5 to 28.5)	-4.9% (-20.6 to 22.3)	2.64% (1.55 to 4.10)	3.6% (-9.9 to 27.6)	395.4 (224.1 to 653.2)	15.7% (-4.8 to 48.0)	1.31% (0.74 to 2.12)	27.9% (7.9 to 60.4)
Bermuda	14.4 (8.1 to 22.2)	-56.0% (-62.8 to -44.5)	3.24% (1.87 to 4.89)	-16.0% (-26.1 to 0.5)	272.6 (162.0 to 431.3)	-49.4% (-58.5 to -33.5)	1.42% (0.84 to 2.19)	-23.2% (-34.3 to -2.2)
Cuba	16.5 (8.6 to 26.6)	-31.5% (-43.2 to -13.5)	2.98% (1.58 to 4.83)	-12.0% (-22.2 to 5.5)	333.9 (182.7 to 564.1)	-19.9% (-34.0 to -0.1)	1.49% (0.83 to 2.39)	0.4% (-12.9 to 21.2)
Dominica	21.1 (11.8 to 33.4)	-11.7% (-26.0 to 10.5)	2.46% (1.43 to 3.73)	-2.5% (-14.4 to 17.3)	428.8 (239.4 to 710.6)	1.8% (-15.0 to 25.2)	1.29% (0.73 to 2.07)	5.2% (-8.4 to 25.5)
Dominican Republic	27.7 (14.6 to 46.7)	41.8% (13.9 to 79.2)	3.62% (1.98 to 5.74)	41.3% (28.3 to 59.0)	557.1 (285.3 to 968.6)	58.9% (26.2 to 99.7)	1.73% (0.94 to 2.82)	85.7% (65.3 to 111.5)
Grenada	24.2 (13.8 to 38.0)	-15.0% (-26.3 to 3.9)	2.94% (1.69 to 4.52)	7.9% (-5.1 to 30.8)	482.7 (259.2 to 801.5)	-5.1% (-19.2 to 15.8)	1.58% (0.89 to 2.61)	18.1% (2.0 to 43.2)
Guyana	35.1 (19.2 to 58.4)	-3.9% (-23.8 to 25.1)	3.11% (1.75 to 4.98)	26.3% (12.1 to 50.9)	729.6 (389.1 to 1273.9)	4.3% (-17.2 to 34.9)	1.67% (0.89 to 2.81)	34.6% (17.4 to 61.5)
Haiti	27.6 (13.9 to 48.2)	-10.8% (-29.1 to 12.4)	2.17% (1.10 to 3.67)	26.1% (11.5 to 47.8)	533.8 (272.6 to 999.2)	-8.6% (-25.9 to 13.8)	1.00% (0.51 to 1.76)	36.9% (20.5 to 59.6)
Jamaica	24.2 (14.5 to 36.4)	8.6% (-11.6 to 38.0)	3.80% (2.37 to 5.48)	14.2% (0.9 to 32.5)	507.5 (299.4 to 786.7)	23.6% (2.6 to 51.9)	1.89% (1.14 to 2.89)	30.1% (16.1 to 49.2)
Puerto Rico	13.7 (8.0 to 21.1)	-43.1% (-57.1 to -23.7)	2.99% (1.88 to 4.42)	-8.7% (-23.4 to 15.4)	354.5 (205.7 to 564.1)	-21.8% (-39.7 to 2.4)	1.60% (0.97 to 2.42)	5.6% (-14.3 to 34.3)
Saint Kitts and Nevis	24.7 (13.8 to 39.5)	-39.1% (-47.8 to -24.9)	2.77% (1.59 to 4.32)	-7.0% (-17.4 to 12.1)	479.4 (268.2 to 768.4)	-30.9% (-42.0 to -13.6)	1.48% (0.82 to 2.37)	-2.8% (-16.4 to 18.1)
Saint Lucia	19.9 (11.3 to 31.0)	-37.0% (-46.6 to -23.5)	2.81% (1.66 to 4.26)	-8.0% (-18.7 to 8.5)	434.7 (238.3 to 709.0)	-21.7% (-33.8 to -5.7)	1.51% (0.86 to 2.44)	0.5% (-12.0 to 17.6)
Saint Vincent and the Grenadines	28.8 (16.3 to 45.1)	-11.8% (-22.6 to 4.1)	3.43% (1.98 to 5.23)	3.0% (-7.4 to 19.8)	559.3 (310.4 to 908.2)	-1.2% (-14.8 to 16.7)	1.76% (0.99 to 2.85)	15.4% (2.3 to 35.4)
Suriname	20.1 (11.2 to 33.1)	-10.4% (-25.5 to 15.3)	2.59% (1.49 to 4.04)	9.1% (-4.2 to 34.5)	468.5 (261.0 to 781.7)	9.2% (-10.3 to 42.6)	1.38% (0.76 to 2.25)	33.6% (13.2 to 67.3)
Trinidad and Tobago	32.9 (18.7 to 50.8)	-26.3% (-43.2 to -3.9)	4.76% (2.94 to 6.96)	9.1% (-2.8 to 27.9)	733.6 (412.7 to 1184.9)	-17.3% (-34.6 to 6.2)	2.51% (1.48 to 3.82)	6.3% (-7.7 to 24.9)
United States Virgin Islands	18.3 (9.5 to 31.5)	-3.5% (-22.4 to 29.3)	2.35% (1.22 to 4.06)	3.3% (-13.5 to 36.6)	311.2 (167.9 to 552.9)	2.6% (-18.7 to 40.3)	1.10% (0.59 to 1.96)	15.8% (-6.2 to 55.8)
Central Europe	13.2 (6.8 to 23.8)	-31.1% (-38.7 to -21.3)	2.02% (1.02 to 3.66)	5.2% (-3.3 to 19.0)	204.0 (109.9 to 378.6)	-29.2% (-38.7 to -13.2)	0.85% (0.45 to 1.55)	4.0% (-7.8 to 25.6)
Albania	7.7 (3.3 to 16.0)	3.5% (-20.0 to 30.0)	1.34% (0.60 to 2.69)	49.3% (30.4 to 70.4)	116.8 (53.3 to 246.0)	4.8% (-16.8 to 30.0)	0.51% (0.24 to 1.03)	51.1% (30.1 to 75.8)
Bosnia and Herzegovina	13.4 (6.6 to 25.2)	12.4% (-9.9 to 52.3)	1.96% (0.96 to 3.65)	38.3% (16.9 to 75.9)	217.0 (111.6 to 416.8)	14.0% (-11.3 to 58.9)	0.90% (0.46 to 1.71)	44.2% (18.1 to 93.4)
Bulgaria	16.8 (8.1 to 32.3)	-20.3% (-35.5 to 1.5)	1.88% (0.91 to 3.62)	0.0% (-14.0 to 20.2)	240.8 (120.3 to 472.2)	-17.5% (-32.6 to 3.0)	0.82% (0.42 to 1.57)	2.1% (-10.4 to 19.2)
Croatia	11.0 (5.4 to 20.4)	-31.4% (-44.1 to -14.1)	1.87% (0.94 to 3.38)	8.0% (-4.1 to 27.8)	159.9 (85.9 to 293.6)	-28.8% (-42.1 to -7.8)	0.73% (0.39 to 1.36)	2.5% (-12.5 to 25.8)
Czechia	13.2 (6.7 to 24.2)	-38.6% (-51.8 to -21.3)	2.43% (1.24 to 4.39)	13.3% (-6.9 to 40.4)	213.8 (116.5 to 389.3)	-33.1% (-48.8 to -9.2)	1.01% (0.56 to 1.79)	4.1% (-17.4 to 36.3)
Hungary	10.4 (5.1 to 19.7)	-27.4% (-40.3 to -10.8)	1.55% (0.77 to 2.93)	17.8% (1.0 to 37.7)	159.2 (82.1 to 301.9)	-27.1% (-40.8 to -7.0)	0.65% (0.35 to 1.22)	12.0% (-6.7 to 36.5)

TABLE S1. Continue.

1	2	3	4	5	6	7	8	9
North Macedonia	20.3 (9.8 to 39.6)	22.0% (1.8 to 48.1)	2.19% (1.08 to 4.26)	36.1% (19.7 to 56.2)	289.7 (145.9 to 550.2)	15.2% (-4.4 to 41.4)	1.04% (0.54 to 1.99)	49.5% (30.3 to 74.0)
Montenegro	10.8 (5.1 to 20.8)	19.4% (3.1 to 42.2)	1.44% (0.69 to 2.71)	19.0% (7.1 to 36.1)	181.8 (92.7 to 338.3)	20.7% (4.7 to 41.5)	0.73% (0.38 to 1.36)	34.8% (21.4 to 52.5)
Poland	11.9 (6.1 to 20.8)	-46.0% (-54.6 to - 34.0)	2.05% (1.05 to 3.66)	-11.3% (-21.3 to 6.0)	198.3 (106.4 to 342.5)	-44.4% (-54.0 to - 28.7)	0.87% (0.47 to 1.53)	-15.2% (-27.3 to 8.7)
Romania	15.8 (7.8 to 29.0)	-23.0% (-35.8 to - 7.4)	2.21% (1.09 to 3.99)	12.8% (-0.4 to 30.7)	231.1 (119.8 to 430.7)	-18.0% (-32.1 to - 0.2)	0.89% (0.46 to 1.61)	18.3% (3.7 to 38.2)
Serbia	17.4 (8.4 to 33.1)	-1.0% (-16.7 to 20.2)	2.13% (1.05 to 4.02)	21.8% (9.6 to 38.0)	243.1 (123.4 to 456.7)	-5.7% (-21.9 to 15.3)	0.96% (0.49 to 1.77)	30.6% (16.0 to 51.1)
Slovakia	13.8 (6.4 to 26.3)	-26.7% (-39.9 to - 9.4)	2.21% (1.05 to 4.12)	14.7% (2.4 to 33.8)	202.5 (102.1 to 384.7)	-29.9% (-43.1 to - 11.5)	0.87% (0.44 to 1.65)	2.1% (-11.9 to 23.9)
Slovenia	5.5 (2.8 to 9.9)	-45.5% (-60.1 to - 25.1)	1.24% (0.66 to 2.24)	-1.4% (-18.4 to 21.4)	97.9 (53.3 to 177.4)	-37.8% (-54.7 to - 13.1)	0.51% (0.28 to 0.90)	-5.1% (-25.4 to 22.6)
Eastern Europe	17.3 (8.1 to 32.3)	-10.4% (-20.6 to 1.0)	2.06% (0.95 to 3.93)	6.9% (-3.5 to 18.2)	232.6 (108.2 to 473.8)	-11.4% (-21.4 to 0.0)	0.73% (0.34 to 1.47)	4.4% (-5.7 to 14.9)
Belarus	20.1 (8.9 to 38.8)	2.7% (-15.8 to 23.9)	2.56% (1.14 to 4.82)	18.6% (6.7 to 32.1)	265.7 (118.6 to 535.7)	3.3% (-16.2 to 25.8)	0.94% (0.42 to 1.85)	24.3% (11.1 to 37.8)
Estonia	10.2 (4.8 to 19.8)	-50.7% (-62.7 to - 34.4)	1.75% (0.82 to 3.32)	-14.2% (-30.8 to 13.0)	134.8 (67.8 to 260.1)	-49.3% (-61.1 to - 30.9)	0.59% (0.29 to 1.14)	-19.1% (-34.9 to 5.9)
Latvia	22.4 (11.1 to 39.0)	-24.1% (-35.0 to - 8.3)	3.28% (1.66 to 5.55)	11.1% (-1.0 to 29.6)	302.1 (157.6 to 553.4)	-23.2% (-34.7 to - 6.2)	1.16% (0.59 to 2.05)	10.5% (-2.3 to 30.4)
Lithuania	23.3 (11.8 to 40.0)	-20.0% (-32.4 to - 3.5)	3.50% (1.78 to 5.99)	8.7% (-2.0 to 26.4)	301.4 (156.1 to 548.0)	-21.5% (-33.7 to - 4.9)	1.18% (0.60 to 2.11)	5.1% (-5.2 to 21.6)
Republic of Moldova	16.5 (7.2 to 31.7)	-30.0% (-38.8 to - 19.6)	2.22% (0.97 to 4.23)	4.4% (-5.2 to 16.5)	224.7 (103.7 to 446.2)	-24.5% (-33.6 to - 12.5)	0.79% (0.37 to 1.54)	7.6% (-2.6 to 21.5)
Russian Federation	17.4 (8.3 to 32.1)	-12.9% (-24.8 to 2.6)	2.15% (1.04 to 3.98)	9.5% (-2.3 to 24.4)	231.5 (110.3 to 454.9)	-15.5% (-26.6 to 0.0)	0.75% (0.36 to 1.48)	3.9% (-7.3 to 18.1)
Ukraine	15.9 (6.2 to 33.3)	-0.9% (-21.8 to 19.7)	1.61% (0.63 to 3.40)	-1.7% (-20.9 to 15.3)	224.1 (91.3 to 482.6)	3.0% (-17.4 to 24.2)	0.63% (0.26 to 1.35)	2.2% (-15.8 to 18.5)
North Africa and Middle East	34.8 (20.4 to 54.4)	-15.1% (-23.0 to - 5.6)	4.50% (2.64 to 6.89)	25.7% (15.9 to 38.8)	671.9 (389.1 to 1089.5)	-9.6% (-20.1 to 5.5)	2.16% (1.28 to 3.43)	43.3% (28.4 to 65.9)
Afghanistan	46.1 (23.8 to 76.4)	-3.3% (-24.0 to 21.1)	3.21% (1.78 to 5.21)	26.9% (12.1 to 48.0)	909.9 (466.2 to 1605.9)	-1.6% (-23.5 to 28.5)	1.64% (0.87 to 2.86)	53.3% (27.1 to 90.4)
Algeria	42.2 (24.7 to 63.8)	-28.7% (-41.6 to - 12.1)	5.75% (3.39 to 8.63)	19.4% (6.9 to 36.8)	671.7 (396.1 to 1077.4)	-23.6% (-38.2 to - 1.9)	2.49% (1.48 to 3.91)	26.1% (9.0 to 52.2)
Bahrain	47.9 (31.2 to 67.7)	-33.3% (-47.5 to - 10.1)	6.55% (4.32 to 9.04)	13.0% (-4.5 to 44.3)	904.2 (563.5 to 1327.3)	-28.5% (-45.8 to - 0.8)	3.70% (2.39 to 5.28)	11.9% (-10.7 to 49.6)
Egypt	51.8 (26.9 to 85.7)	1.3% (-19.6 to 25.8)	5.06% (2.75 to 8.02)	32.3% (17.0 to 53.1)	976.3 (498.5 to 1705.6)	9.1% (-14.1 to 38.8)	2.86% (1.55 to 4.77)	69.2% (47.7 to 100.7)
Iran (Islamic Republic of)	24.0 (13.1 to 38.6)	-30.4% (-36.7 to - 19.9)	4.03% (2.22 to 6.43)	24.8% (12.8 to 43.7)	433.3 (243.2 to 722.0)	-26.0% (-35.4 to - 6.4)	1.74% (0.97 to 2.87)	38.5% (21.2 to 73.7)
Iraq	49.9 (29.5 to 75.7)	-6.2% (-22.7 to 10.6)	6.09% (3.74 to 8.88)	23.6% (13.9 to 36.3)	929.8 (543.0 to 1460.1)	-6.3% (-24.7 to 13.8)	2.95% (1.77 to 4.53)	38.7% (22.7 to 57.9)
Jordan	24.4 (14.8 to 37.3)	-32.3% (-43.9 to - 16.6)	4.26% (2.60 to 6.42)	11.4% (-0.4 to 30.0)	454.0 (265.9 to 726.3)	-26.2% (-38.6 to - 9.3)	1.93% (1.13 to 3.01)	9.8% (-3.1 to 27.9)
Kuwait	23.3 (14.7 to 34.0)	-34.2% (-44.2 to - 21.0)	5.34% (3.38 to 7.55)	2.3% (-6.2 to 13.8)	559.0 (349.8 to 838.3)	-22.3% (-34.4 to - 7.1)	2.77% (1.74 to 4.02)	14.3% (1.3 to 31.5)
Lebanon	36.9 (20.0 to 57.7)	-19.5% (-34.1 to - 4.0)	5.54% (2.98 to 8.61)	20.5% (0.8 to 39.1)	719.9 (400.7 to 1149.1)	-12.7% (-28.6 to 8.8)	2.79% (1.56 to 4.38)	33.4% (13.7 to 59.9)
Libya	29.7 (17.4 to 46.5)	1.4% (-19.1 to 29.4)	4.55% (2.68 to 6.89)	20.2% (9.5 to 34.0)	643.4 (375.0 to 1034.9)	14.6% (-7.9 to 45.9)	2.29% (1.35 to 3.58)	41.8% (25.0 to 64.1)
Morocco	44.8 (25.1 to 69.9)	11.6% (-7.5 to 32.0)	5.26% (3.02 to 8.05)	42.4% (27.6 to 64.0)	802.8 (451.3 to 1317.5)	13.6% (-6.8 to 38.9)	2.60% (1.48 to 4.20)	71.3% (49.7 to 106.8)

TABLE S1. Continue.

1	2	3	4	5	6	7	8	9
Palestine	44.2 (27.2 to 66.4)	-8.1% (-25.1 to 16.5)	5.55% (3.47 to 8.22)	23.9% (12.3 to 41.7)	785.7 (476.7 to 1226.3)	-6.0% (-24.6 to 20.8)	2.75% (1.70 to 4.28)	33.5% (14.3 to 59.1)
Oman	60.8 (37.1 to 89.5)	-0.7% (-20.7 to 26.5)	6.66% (4.03 to 9.88)	38.7% (19.5 to 70.0)	1008.5 (603.2 to 1536.2)	-5.9% (-27.0 to 25.2)	3.50% (2.12 to 5.32)	43.8% (19.2 to 81.5)
Qatar	64.5 (41.3 to 91.2)	-15.3% (-31.7 to 7.2)	7.36% (4.90 to 10.18)	11.6% (0.0 to 28.7)	1073.3 (689.2 to 1547.8)	-17.8% (-35.4 to 7.6)	4.19% (2.74 to 5.90)	19.3% (2.6 to 42.6)
Saudi Arabia	46.9 (30.8 to 66.5)	-1.3% (-22.9 to 30.2)	6.18% (4.05 to 8.77)	41.6% (23.9 to 71.4)	1041.2 (668.7 to 1500.2)	14.8% (-12.4 to 54.2)	3.64% (2.34 to 5.14)	67.6% (40.3 to 106.8)
Sudan	58.7 (37.1 to 86.6)	-12.2% (-26.0 to 6.4)	6.27% (4.12 to 8.77)	41.4% (28.6 to 58.0)	1176.2 (725.6 to 1775.0)	-10.7% (-27.5 to 12.5)	3.12% (1.97 to 4.59)	68.7% (44.4 to 101.4)
Syrian Arab Republic	51.6 (27.0 to 84.2)	-0.3% (-22.9 to 30.9)	5.92% (3.23 to 9.26)	18.4% (8.9 to 31.7)	874.7 (466.7 to 1476.5)	-4.9% (-27.3 to 27.2)	2.85% (1.52 to 4.70)	26.9% (11.0 to 47.8)
Tunisia	15.3 (6.4 to 30.7)	7.3% (-16.8 to 42.0)	2.52% (1.11 to 4.84)	44.2% (24.2 to 74.8)	260.7 (120.5 to 524.1)	16.7% (-10.1 to 55.0)	1.08% (0.51 to 2.12)	67.9% (40.1 to 109.2)
Turkey	20.2 (11.4 to 31.6)	-28.6% (-44.0 to -8.5)	3.58% (2.11 to 5.52)	20.8% (4.1 to 44.8)	372.0 (215.3 to 600.7)	-30.0% (-45.3 to -9.1)	1.57% (0.92 to 2.51)	25.0% (0.9 to 56.6)
United Arab Emirates	47.6 (29.9 to 69.0)	-34.0% (-46.3 to -19.4)	5.70% (3.78 to 8.12)	0.3% (-11.6 to 13.0)	1026.4 (647.2 to 1493.8)	-23.8% (-38.6 to -6.4)	3.55% (2.32 to 5.08)	4.4% (-7.5 to 19.0)
Yemen	39.4 (20.8 to 67.0)	-3.6% (-21.6 to 21.7)	3.68% (1.95 to 6.01)	32.6% (18.1 to 54.5)	695.5 (360.0 to 1225.3)	-5.2% (-25.0 to 22.1)	1.57% (0.81 to 2.71)	43.6% (22.8 to 73.5)
Central Asia	21.5 (9.8 to 41.4)	29.6% (18.2 to 42.0)	2.16% (0.98 to 4.13)	34.1% (24.6 to 45.6)	316.6 (147.1 to 622.4)	30.7% (16.6 to 48.2)	0.92% (0.42 to 1.82)	61.7% (44.7 to 82.1)
Armenia	15.4 (7.2 to 29.3)	-10.3% (-24.1 to 8.9)	2.12% (1.00 to 4.02)	17.1% (3.6 to 37.3)	221.5 (108.3 to 446.0)	-6.1% (-22.1 to 16.9)	0.86% (0.41 to 1.66)	34.3% (14.7 to 62.7)
Azerbaijan	26.6 (11.5 to 53.4)	91.9% (63.0 to 131.5)	2.42% (1.03 to 4.79)	80.8% (56.9 to 112.5)	354.0 (159.1 to 712.5)	65.1% (35.1 to 102.3)	1.02% (0.45 to 2.06)	117.6% (81.2 to 163.5)
Georgia	13.3 (6.2 to 26.2)	-37.8% (-48.5 to -21.5)	1.64% (0.76 to 3.25)	-16.2% (-26.3 to 2.0)	211.3 (102.6 to 419.7)	-26.7% (-40.9 to -4.0)	0.71% (0.34 to 1.43)	-2.7% (-17.7 to 23.8)
Kazakhstan	22.8 (10.7 to 41.8)	7.0% (-5.7 to 24.3)	2.48% (1.17 to 4.38)	21.7% (11.0 to 37.4)	327.9 (162.5 to 618.5)	4.4% (-9.4 to 24.9)	1.01% (0.50 to 1.87)	32.5% (17.0 to 54.6)
Kyrgyzstan	17.6 (7.4 to 34.8)	38.5% (4.6 to 69.2)	2.17% (0.90 to 4.32)	73.3% (34.7 to 106.6)	233.5 (99.9 to 486.1)	23.3% (-3.8 to 49.5)	0.79% (0.34 to 1.63)	81.1% (41.8 to 115.4)
Mongolia	15.2 (6.1 to 31.2)	-5.0% (-21.9 to 19.6)	1.27% (0.52 to 2.59)	23.1% (8.1 to 47.1)	201.8 (82.1 to 450.6)	-10.6% (-28.3 to 14.4)	0.50% (0.21 to 1.09)	35.4% (19.4 to 61.8)
Tajikistan	20.1 (8.3 to 43.7)	127.4% (88.7 to 180.6)	1.77% (0.73 to 3.69)	97.1% (72.5 to 132.4)	284.1 (120.8 to 617.9)	101.7% (66.6 to 149.9)	0.76% (0.33 to 1.60)	148.7% (113.2 to 191.8)
Turkmenistan	20.8 (9.1 to 41.0)	13.2% (-5.7 to 38.2)	2.29% (1.00 to 4.51)	40.8% (26.5 to 62.7)	320.1 (141.4 to 669.5)	21.9% (0.9 to 50.5)	0.92% (0.41 to 1.87)	64.5% (43.1 to 95.3)
Uzbekistan	37.3 (15.6 to 75.3)	159.0% (118.8 to 207.0)	2.72% (1.14 to 5.44)	76.9% (53.5 to 105.1)	499.4 (216.6 to 1027.7)	146.6% (98.5 to 195.9)	1.25% (0.54 to 2.54)	142.6% (100.3 to 186.3)
South Asia	12.3 (6.2 to 22.2)	-2.3% (-16.3 to 13.5)	1.34% (0.68 to 2.41)	64.5% (45.1 to 89.6)	205.8 (103.2 to 389.9)	-6.5% (-19.0 to 8.0)	0.53% (0.27 to 1.01)	59.7% (40.5 to 85.5)
Bangladesh	11.3 (5.4 to 21.1)	8.3% (-13.9 to 33.6)	1.59% (0.79 to 2.85)	129.0% (95.5 to 168.3)	177.4 (88.5 to 339.5)	5.2% (-14.9 to 28.7)	0.58% (0.29 to 1.12)	133.4% (99.4 to 173.9)
Bhutan	17.1 (9.0 to 29.2)	29.8% (-0.1 to 70.9)	2.09% (1.08 to 3.51)	121.1% (73.7 to 182.2)	283.1 (151.1 to 511.0)	25.3% (-2.2 to 62.5)	0.86% (0.45 to 1.50)	147.6% (100.1 to 208.9)
India	11.7 (5.8 to 21.0)	-6.2% (-22.1 to 11.4)	1.29% (0.66 to 2.37)	64.8% (42.4 to 94.4)	191.5 (96.0 to 368.2)	-12.6% (-26.6 to 3.7)	0.51% (0.25 to 0.96)	53.1% (31.3 to 80.7)
Nepal	9.9 (4.8 to 19.4)	36.9% (5.2 to 82.4)	1.04% (0.50 to 1.96)	119.7% (78.4 to 177.2)	167.8 (82.2 to 330.0)	31.9% (3.3 to 70.1)	0.47% (0.23 to 0.91)	157.0% (110.0 to 218.5)
Pakistan	20.9 (10.6 to 36.6)	40.8% (17.0 to 74.4)	1.82% (0.96 to 3.16)	68.0% (47.1 to 96.1)	399.8 (198.7 to 729.8)	45.2% (21.7 to 76.6)	0.83% (0.41 to 1.50)	85.8% (60.0 to 118.0)
Southeast Asia	10.2 (5.0 to 19.5)	15.4% (2.4 to 31.2)	1.26% (0.61 to 2.40)	63.2% (49.3 to 81.8)	192.6 (93.7 to 364.2)	21.0% (6.8 to 38.7)	0.61% (0.30 to 1.17)	88.5% (70.0 to 113.1)

TABLE S1. Continue.

1	2	3	4	5	6	7	8	9
Cambodia	8.3 (3.7 to 17.2)	10.2% (-11.0 to 32.2)	0.82% (0.36 to 1.66)	74.5% (44.8 to 109.8)	141.9 (66.3 to 293.3)	9.1% (-10.8 to 34.0)	0.38% (0.18 to 0.77)	103.7% (68.4 to 147.4)
Indonesia	16.1 (7.8 to 31.0)	51.3% (27.3 to 78.4)	1.69% (0.82 to 3.24)	92.8% (73.5 to 120.0)	293.3 (141.1 to 559.2)	47.6% (25.3 to 74.0)	0.86% (0.41 to 1.63)	127.4% (101.1 to 161.7)
Lao People's Democratic Republic	10.0 (4.3 to 21.5)	15.2% (-8.8 to 48.9)	0.97% (0.41 to 1.98)	108.1% (70.3 to 156.7)	173.6 (77.4 to 365.0)	10.7% (-11.2 to 41.2)	0.44% (0.20 to 0.92)	136.7% (96.3 to 190.7)
Malaysia	16.6 (7.9 to 29.5)	-3.0% (-20.9 to 19.5)	2.23% (1.08 to 3.92)	13.3% (0.5 to 31.1)	342.9 (169.3 to 620.7)	2.4% (-14.4 to 23.8)	1.28% (0.66 to 2.25)	25.3% (13.5 to 43.4)
Maldives	12.5 (6.0 to 23.4)	-40.9% (-52.9 to -24.9)	2.33% (1.10 to 4.17)	35.5% (15.1 to 64.4)	229.6 (110.6 to 432.8)	-40.6% (-52.2 to -22.1)	1.03% (0.50 to 1.92)	28.3% (5.5 to 63.8)
Mauritius	20.9 (10.8 to 35.6)	-7.5% (-32.2 to 50.8)	3.07% (1.66 to 4.93)	34.0% (3.3 to 107.8)	442.5 (225.5 to 774.7)	1.4% (-28.1 to 63.1)	1.58% (0.81 to 2.69)	31.0% (-3.6 to 106.6)
Myanmar	11.1 (5.2 to 22.3)	-0.5% (-19.1 to 24.0)	1.11% (0.50 to 2.19)	73.0% (45.5 to 110.7)	191.5 (92.8 to 372.3)	-4.9% (-23.8 to 21.3)	0.49% (0.23 to 0.97)	88.5% (54.4 to 133.7)
Philippines	4.8 (1.9 to 10.9)	8.9% (-11.5 to 31.8)	0.56% (0.23 to 1.28)	28.2% (12.5 to 48.1)	86.7 (36.5 to 194.3)	29.3% (5.7 to 58.8)	0.26% (0.11 to 0.58)	64.9% (41.4 to 94.5)
Sri Lanka	9.0 (3.9 to 18.5)	-3.1% (-27.6 to 48.2)	1.46% (0.65 to 2.85)	57.4% (26.7 to 122.1)	160.8 (72.3 to 320.7)	7.7% (-20.1 to 62.8)	0.65% (0.30 to 1.27)	68.2% (31.7 to 141.3)
Seychelles	10.1 (4.8 to 19.4)	-2.0% (-15.9 to 23.1)	1.25% (0.61 to 2.35)	15.7% (0.9 to 43.9)	211.1 (104.2 to 395.0)	13.5% (-7.7 to 52.2)	0.71% (0.36 to 1.30)	33.2% (10.1 to 76.5)
Thailand	5.4 (2.5 to 10.4)	-22.8% (-41.8 to 2.5)	1.01% (0.49 to 1.90)	22.7% (5.6 to 48.3)	111.8 (54.3 to 206.8)	-7.6% (-28.2 to 21.2)	0.46% (0.23 to 0.85)	35.7% (15.3 to 64.3)
Timor-Leste	10.6 (4.4 to 22.5)	40.3% (10.3 to 73.4)	1.09% (0.44 to 2.22)	93.5% (60.4 to 128.7)	172.2 (78.1 to 365.4)	36.5% (10.1 to 66.1)	0.47% (0.21 to 0.98)	141.5% (103.3 to 184.9)
Viet Nam	7.7 (3.4 to 16.9)	17.0% (-5.0 to 47.1)	1.01% (0.44 to 2.21)	52.6% (27.0 to 85.8)	124.1 (57.0 to 259.0)	19.1% (-3.7 to 49.5)	0.45% (0.21 to 0.96)	67.1% (41.2 to 99.6)
East Asia	10.1 (4.6 to 19.2)	6.6% (-17.6 to 34.5)	1.59% (0.72 to 3.01)	88.3% (50.0 to 125.6)	145.7 (70.1 to 277.7)	-0.7% (-20.6 to 22.1)	0.65% (0.31 to 1.25)	82.1% (49.3 to 116.7)
China	10.2 (4.6 to 19.4)	7.5% (-18.0 to 36.8)	1.61% (0.72 to 3.05)	91.2% (51.4 to 131.1)	146.1 (69.8 to 279.9)	-0.4% (-21.1 to 22.9)	0.66% (0.31 to 1.27)	84.1% (50.8 to 120.0)
Democratic People's Republic of Korea	9.6 (4.3 to 19.2)	8.3% (-12.7 to 36.9)	1.17% (0.52 to 2.33)	39.2% (16.9 to 69.3)	154.2 (71.5 to 322.2)	8.6% (-11.2 to 33.3)	0.52% (0.24 to 1.08)	56.9% (34.1 to 85.7)
Taiwan (Province of China)	7.6 (4.1 to 12.8)	-21.9% (-41.7 to 8.8)	1.59% (0.90 to 2.53)	20.2% (-3.7 to 59.3)	140.3 (76.5 to 248.7)	-12.4% (-31.0 to 18.5)	0.71% (0.39 to 1.23)	19.6% (-2.7 to 54.5)
Oceania	24.3 (12.3 to 43.7)	28.4% (7.9 to 55.2)	1.93% (1.00 to 3.32)	39.2% (24.2 to 55.3)	512.1 (255.7 to 933.3)	33.1% (11.4 to 61.7)	1.04% (0.52 to 1.84)	46.7% (30.4 to 64.6)
American Samoa	29.6 (16.7 to 47.2)	17.9% (1.6 to 40.1)	3.34% (1.95 to 5.23)	31.3% (18.6 to 46.4)	735.9 (412.4 to 1227.8)	32.6% (14.2 to 54.5)	2.25% (1.29 to 3.70)	42.8% (28.2 to 60.9)
Federated States of Micronesia	47.1 (26.1 to 79.4)	45.6% (10.7 to 93.4)	3.19% (1.78 to 5.18)	59.9% (34.5 to 91.3)	996.4 (516.2 to 1798.7)	49.0% (9.8 to 102.1)	2.01% (1.06 to 3.48)	73.0% (39.2 to 112.9)
Cook Islands	24.1 (13.6 to 38.5)	2.9% (-15.4 to 26.9)	3.38% (1.97 to 5.29)	57.6% (36.5 to 81.6)	545.3 (291.3 to 922.7)	15.0% (-4.6 to 41.8)	2.02% (1.14 to 3.29)	63.0% (41.3 to 87.6)
Nauru	43.8 (23.1 to 76.4)	30.4% (12.0 to 55.0)	2.89% (1.55 to 4.97)	38.9% (23.3 to 60.2)	902.4 (455.1 to 1664.3)	37.2% (15.7 to 65.4)	1.74% (0.88 to 3.11)	47.4% (29.2 to 71.5)
Niue	32.8 (18.2 to 55.8)	22.7% (0.5 to 54.4)	3.37% (1.89 to 5.54)	40.6% (25.1 to 61.0)	706.8 (378.7 to 1255.2)	37.6% (12.2 to 72.2)	1.97% (1.06 to 3.35)	53.8% (34.3 to 78.0)
Palau	32.6 (17.9 to 55.0)	27.7% (-2.5 to 68.3)	2.75% (1.54 to 4.50)	44.9% (27.1 to 71.4)	701.8 (368.6 to 1264.8)	37.0% (4.7 to 79.1)	1.68% (0.89 to 2.89)	56.5% (35.5 to 83.4)
Fiji	48.8 (26.0 to 82.1)	46.2% (13.2 to 94.6)	4.21% (2.39 to 6.76)	61.9% (38.4 to 92.9)	1004.4 (512.3 to 1761.5)	48.9% (12.8 to 96.8)	2.49% (1.32 to 4.20)	64.2% (40.5 to 92.0)
Guam	13.9 (6.6 to 25.5)	-27.2% (-42.5 to -9.2)	2.26% (1.10 to 4.09)	2.4% (-16.4 to 24.8)	292.0 (147.4 to 553.7)	-8.5% (-27.2 to 12.6)	1.08% (0.55 to 2.01)	-0.2% (-17.2 to 19.9)
Kiribati	39.1 (20.3 to 68.4)	28.4% (3.1 to 62.7)	2.27% (1.22 to 3.86)	61.4% (40.3 to 90.5)	868.4 (430.0 to 1609.7)	23.8% (-1.6 to 59.0)	1.45% (0.74 to 2.56)	66.3% (41.7 to 94.2)

TABLE S1. Continue.

1	2	3	4	5	6	7	8	9
Marshall Islands	37.4 (19.8 to 65.1)	24.5% (-0.4 to 54.6)	2.79% (1.50 to 4.66)	43.7% (27.4 to 65.1)	869.4 (439.2 to 1572.2)	36.8% (10.5 to 70.7)	1.85% (0.96 to 3.21)	55.7% (37.4 to 79.6)
Northern Mariana Islands	21.1 (11.6 to 35.3)	12.4% (-7.3 to 36.6)	2.50% (1.37 to 4.10)	32.6% (15.6 to 49.2)	456.0 (247.4 to 803.2)	23.1% (3.0 to 46.5)	1.49% (0.81 to 2.50)	38.8% (23.2 to 55.1)
Papua New Guinea	19.1 (9.4 to 35.0)	37.8% (12.2 to 71.0)	1.51% (0.75 to 2.72)	45.5% (27.0 to 66.7)	404.6 (194.0 to 772.9)	39.5% (14.1 to 72.2)	0.80% (0.38 to 1.47)	55.5% (35.0 to 78.2)
Samoa	29.6 (16.1 to 50.7)	14.0% (-8.7 to 43.0)	3.01% (1.68 to 4.98)	37.0% (22.2 to 57.5)	620.7 (332.6 to 1089.5)	22.1% (-3.3 to 55.0)	1.77% (0.98 to 3.00)	47.5% (30.8 to 69.9)
Solomon Islands	35.8 (17.1 to 68.0)	43.2% (12.2 to 85.9)	1.87% (0.89 to 3.57)	61.9% (38.0 to 99.9)	787.8 (367.1 to 1567.8)	43.2% (8.8 to 91.3)	1.22% (0.56 to 2.38)	68.6% (40.4 to 110.3)
Tokelau	27.8 (15.0 to 48.1)	22.5% (-3.4 to 55.7)	3.12% (1.68 to 5.10)	57.2% (36.4 to 82.9)	576.8 (297.2 to 1034.3)	26.3% (0.0 to 61.1)	1.80% (0.96 to 3.05)	62.3% (40.8 to 87.9)
Tonga	22.9 (12.6 to 38.4)	19.3% (-2.2 to 47.2)	2.78% (1.58 to 4.45)	32.4% (20.0 to 47.2)	488.7 (262.3 to 850.8)	22.9% (1.6 to 51.5)	1.59% (0.86 to 2.67)	38.7% (26.2 to 54.3)
Tuvalu	31.3 (16.4 to 56.6)	24.9% (-2.5 to 64.1)	2.70% (1.45 to 4.61)	67.8% (46.1 to 96.4)	632.2 (322.0 to 1198.2)	25.7% (-0.7 to 65.3)	1.56% (0.80 to 2.75)	85.3% (61.1 to 115.9)
Vanuatu	19.6 (8.7 to 40.2)	36.9% (8.8 to 81.9)	1.47% (0.67 to 2.81)	53.0% (30.7 to 87.1)	372.9 (169.3 to 777.9)	40.1% (8.9 to 86.5)	0.80% (0.37 to 1.60)	58.6% (33.8 to 95.3)
High-income Asia Pacific	4.3 (2.1 to 7.5)	-57.8% (-64.6 to -46.6)	1.28% (0.62 to 2.23)	-24.7% (-37.0 to -4.7)	90.5 (45.0 to 163.2)	-45.0% (-54.1 to -28.4)	0.56% (0.29 to 1.02)	-20.5% (-32.8 to 2.1)
Brunei Darussalam	21.0 (10.7 to 36.2)	-26.5% (-36.9 to -11.6)	2.41% (1.23 to 4.14)	4.5% (-7.7 to 20.8)	403.7 (202.6 to 720.8)	-23.6% (-34.0 to -9.3)	1.45% (0.73 to 2.58)	3.3% (-8.5 to 20.0)
Japan	3.9 (1.8 to 6.9)	-58.3% (-66.0 to -47.8)	1.20% (0.57 to 2.11)	-31.0% (-43.7 to -13.5)	84.0 (42.0 to 151.9)	-43.3% (-52.6 to -28.7)	0.53% (0.27 to 0.96)	-25.3% (-36.9 to -7.4)
Singapore	5.3 (2.4 to 9.7)	-62.5% (-69.2 to -53.6)	1.64% (0.74 to 3.00)	-16.8% (-31.8 to 3.0)	115.0 (56.7 to 210.2)	-57.1% (-65.2 to -44.0)	0.77% (0.38 to 1.40)	-27.3% (-40.3 to -5.8)
Republic of Korea	6.7 (3.5 to 11.5)	-59.7% (-67.4 to -43.4)	1.71% (0.90 to 2.94)	-4.4% (-23.3 to 34.6)	120.3 (61.2 to 213.0)	-54.6% (-64.8 to -33.9)	0.70% (0.36 to 1.24)	-14.6% (-33.6 to 22.8)
High-income North America	6.0 (2.7 to 12.2)	-55.3% (-63.3 to -44.2)	1.17% (0.52 to 2.35)	-42.5% (-52.8 to -28.3)	130.4 (64.3 to 247.6)	-43.1% (-54.6 to -22.0)	0.51% (0.25 to 1.00)	-35.4% (-48.3 to -11.7)
Canada	7.2 (3.5 to 12.7)	-44.3% (-51.8 to -30.6)	1.75% (0.86 to 3.08)	-17.1% (-28.1 to 3.8)	123.5 (64.9 to 222.7)	-36.1% (-46.8 to -16.3)	0.63% (0.33 to 1.11)	-21.2% (-34.5 to 2.9)
Greenland	9.6 (4.3 to 17.6)	-40.9% (-51.9 to -24.1)	1.19% (0.54 to 2.19)	-5.0% (-18.0 to 17.5)	164.9 (79.5 to 317.9)	-34.9% (-47.4 to -13.6)	0.52% (0.25 to 0.99)	-0.1% (-14.4 to 26.5)
United States of America	5.9 (2.6 to 12.1)	-56.6% (-65.5 to -45.3)	1.11% (0.49 to 2.29)	-45.0% (-56.5 to -30.9)	131.1 (64.6 to 251.3)	-43.8% (-56.1 to -22.3)	0.50% (0.25 to 0.99)	-36.7% (-50.2 to -12.9)
Western Europe	8.6 (4.5 to 14.2)	-50.1% (-55.1 to -41.3)	2.03% (1.08 to 3.38)	-20.0% (-28.1 to -5.9)	158.1 (88.5 to 268.6)	-41.1% (-49.8 to -24.2)	0.82% (0.46 to 1.39)	-20.5% (-31.3 to 1.9)
Andorra	7.9 (4.2 to 13.1)	-26.8% (-45.8 to 0.5)	1.90% (1.03 to 3.05)	-3.7% (-17.2 to 16.7)	149.2 (82.7 to 249.5)	-13.0% (-35.0 to 18.7)	0.79% (0.45 to 1.27)	2.1% (-15.1 to 27.6)
Austria	9.9 (5.0 to 16.8)	-45.5% (-55.3 to -33.4)	2.36% (1.17 to 3.99)	-8.4% (-25.4 to 12.2)	155.8 (82.8 to 271.0)	-40.4% (-49.9 to -24.4)	0.82% (0.43 to 1.41)	-16.9% (-29.8 to 4.8)
Belgium	8.7 (4.7 to 13.8)	-52.0% (-57.3 to -43.4)	1.93% (1.05 to 3.06)	-25.8% (-33.7 to -12.1)	165.5 (94.6 to 274.7)	-41.1% (-49.8 to -24.7)	0.82% (0.46 to 1.34)	-23.4% (-34.0 to -2.2)
Cyprus	13.0 (6.5 to 23.0)	-51.4% (-59.2 to -39.9)	2.51% (1.25 to 4.37)	-13.7% (-26.7 to 6.0)	199.3 (98.8 to 371.8)	-46.0% (-54.2 to -34.2)	1.01% (0.50 to 1.84)	-23.3% (-33.7 to -7.5)
Denmark	8.4 (4.3 to 14.2)	-53.7% (-62.3 to -36.2)	1.82% (0.92 to 3.03)	-26.3% (-40.2 to 1.4)	137.6 (74.3 to 235.7)	-49.1% (-60.3 to -23.4)	0.69% (0.38 to 1.19)	-29.5% (-44.7 to 5.8)
Finland	10.4 (4.4 to 18.9)	-43.8% (-50.8 to -31.2)	2.42% (1.01 to 4.36)	-5.2% (-16.9 to 15.4)	174.6 (85.9 to 315.0)	-40.4% (-49.8 to -16.9)	0.90% (0.43 to 1.63)	-16.6% (-29.9 to 16.2)
France	7.9 (4.6 to 12.0)	-46.0% (-52.1 to -36.6)	2.03% (1.18 to 3.09)	-12.8% (-22.6 to 2.1)	138.0 (82.5 to 218.1)	-37.6% (-45.8 to -23.6)	0.74% (0.44 to 1.17)	-16.3% (-26.8 to 2.0)
Germany	8.4 (3.8 to 15.7)	-49.1% (-55.2 to -38.6)	1.82% (0.83 to 3.41)	-20.2% (-29.7 to -3.4)	144.1 (71.2 to 273.9)	-40.8% (-50.0 to -23.3)	0.72% (0.35 to 1.34)	-21.1% (-33.3 to 2.7)

TABLE S1. Continue.

1	2	3	4	5	6	7	8	9
Greece	7.8 (3.3 to 15.3)	-31.9% (-42.9 to -15.3)	1.66% (0.70 to 3.20)	-5.7% (-21.2 to 16.9)	125.2 (57.3 to 254.6)	-21.8% (-35.4 to 1.4)	0.62% (0.28 to 1.27)	-5.4% (-21.9 to 21.9)
Iceland	8.2 (4.0 to 14.0)	-50.9% (-57.0 to -40.0)	2.35% (1.13 to 3.95)	-17.0% (-26.9 to 0.8)	149.8 (80.2 to 256.8)	-42.2% (-52.4 to -19.5)	0.86% (0.46 to 1.47)	-22.5% (-35.0 to 6.3)
Ireland	12.8 (7.2 to 19.6)	-56.9% (-61.7 to -49.3)	2.97% (1.66 to 4.53)	-20.3% (-29.2 to -6.7)	223.2 (131.6 to 348.1)	-51.8% (-59.6 to -36.4)	1.16% (0.68 to 1.80)	-31.6% (-42.4 to -9.4)
Israel	9.7 (5.5 to 15.3)	-49.3% (-58.7 to -29.4)	2.48% (1.42 to 3.90)	-16.9% (-32.0 to 15.6)	175.5 (102.7 to 286.1)	-42.2% (-55.3 to -16.1)	0.99% (0.57 to 1.58)	-21.3% (-39.1 to 13.6)
Italy	9.5 (5.2 to 15.1)	-50.1% (-55.5 to -44.0)	2.45% (1.35 to 3.87)	-17.4% (-26.3 to -7.4)	172.8 (99.4 to 283.7)	-42.9% (-50.3 to -31.1)	0.95% (0.55 to 1.56)	-20.1% (-30.1 to -3.8)
Luxembourg	7.2 (3.4 to 12.3)	-60.2% (-67.4 to -50.1)	1.84% (0.90 to 3.15)	-23.8% (-35.7 to -6.2)	142.1 (74.9 to 245.6)	-45.7% (-57.6 to -19.6)	0.76% (0.41 to 1.30)	-19.8% (-35.6 to 16.7)
Malta	17.7 (10.5 to 25.9)	-50.1% (-55.8 to -42.3)	4.42% (2.64 to 6.32)	-14.4% (-22.1 to -3.1)	337.2 (208.4 to 504.5)	-43.4% (-50.8 to -31.6)	1.78% (1.10 to 2.64)	-25.4% (-33.9 to -11.5)
Monaco	11.3 (6.0 to 18.3)	-35.3% (-48.4 to -14.2)	2.26% (1.24 to 3.59)	-23.4% (-34.2 to -6.0)	202.3 (113.8 to 326.3)	-23.9% (-41.0 to 3.9)	0.96% (0.55 to 1.53)	-15.3% (-29.1 to 9.7)
Netherlands	4.4 (2.0 to 8.8)	-46.0% (-56.0 to -29.7)	1.00% (0.44 to 1.98)	-21.2% (-35.8 to 2.9)	70.8 (32.5 to 136.6)	-42.7% (-55.2 to -21.6)	0.38% (0.18 to 0.72)	-26.8% (-42.4 to -0.9)
Norway	8.0 (4.2 to 13.4)	-53.6% (-60.2 to -39.3)	2.03% (1.05 to 3.38)	-23.3% (-34.3 to -0.1)	149.9 (85.2 to 249.9)	-47.8% (-58.2 to -24.3)	0.82% (0.45 to 1.35)	-28.0% (-42.1 to 4.0)
Portugal	11.4 (6.4 to 18.1)	-54.9% (-63.1 to -40.6)	2.59% (1.47 to 4.10)	-17.3% (-32.5 to 8.3)	221.5 (130.7 to 345.3)	-42.1% (-54.2 to -22.6)	1.13% (0.67 to 1.79)	-11.4% (-28.4 to 18.3)
San Marino	8.6 (4.2 to 14.9)	-28.5% (-50.3 to -2.2)	2.07% (1.13 to 3.29)	-11.3% (-21.2 to 3.5)	159.1 (86.2 to 274.6)	-13.5% (-37.0 to 16.6)	0.83% (0.48 to 1.35)	-3.3% (-19.2 to 17.9)
Spain	7.6 (4.1 to 12.5)	-51.0% (-58.8 to -38.3)	1.98% (1.05 to 3.20)	-18.0% (-31.4 to 4.0)	158.7 (88.1 to 265.4)	-37.3% (-48.7 to -19.0)	0.88% (0.49 to 1.46)	-12.1% (-26.7 to 13.0)
Sweden	7.3 (3.3 to 13.8)	-51.2% (-57.5 to -38.7)	1.85% (0.84 to 3.47)	-25.8% (-35.4 to -6.9)	118.1 (57.2 to 219.3)	-47.4% (-56.1 to -28.2)	0.66% (0.32 to 1.23)	-31.9% (-42.9 to -7.2)
Switzerland	7.8 (4.0 to 12.9)	-51.5% (-57.0 to -42.3)	2.22% (1.15 to 3.64)	-18.8% (-28.2 to -3.0)	134.4 (75.3 to 223.8)	-45.1% (-52.9 to -29.2)	0.77% (0.42 to 1.25)	-22.4% (-33.0 to -2.0)
United Kingdom	9.7 (5.3 to 16.0)	-54.6% (-59.5 to -45.1)	2.09% (1.13 to 3.43)	-30.1% (-37.6 to -15.4)	202.0 (115.8 to 337.4)	-42.7% (-53.1 to -18.9)	0.97% (0.55 to 1.59)	-25.9% (-38.3 to 4.9)
Australasia	9.3 (5.0 to 15.0)	-49.2% (-57.3 to -34.2)	2.34% (1.27 to 3.81)	-16.9% (-30.0 to 7.9)	166.6 (95.2 to 272.4)	-40.6% (-52.9 to -16.3)	0.85% (0.47 to 1.39)	-20.5% (-36.2 to 12.1)
Australia	9.1 (4.9 to 14.6)	-51.6% (-59.6 to -36.6)	2.33% (1.27 to 3.77)	-20.7% (-33.9 to 4.8)	165.3 (94.9 to 270.5)	-41.9% (-54.1 to -18.0)	0.85% (0.48 to 1.39)	-22.9% (-38.3 to 9.0)
New Zealand	10.4 (5.4 to 17.4)	-35.1% (-46.7 to -13.4)	2.41% (1.24 to 4.02)	5.5% (-12.7 to 39.9)	173.9 (94.3 to 294.2)	-33.2% (-46.8 to -5.9)	0.84% (0.45 to 1.42)	-7.7% (-26.2 to 29.8)

Abbreviations: DALYs = disability-adjusted life years; PAF = population attributable fraction; SDI = Socio-demographic Index
